# PRMT5 upregulates KCNMB4 expression via histone methylation to promote paclitaxel resistance in advanced nasopharyngeal carcinoma

**DOI:** 10.1038/s41419-025-08190-y

**Published:** 2026-01-09

**Authors:** Lizhen Liu, Sailan Liu, Yali Wang, Peili Wang, Guixiang Zhong, Jing Han Hong, Rong Xiao, Yaoyu Guo, Fang Zhu, Jing Hao, JianFeng Chen, Hai-Qiang Mai, Jing Tan

**Affiliations:** 1 Medical Research Institute, Guangdong Provincial People’s Hospital (Guangdong Academy of Medical Sciences), Southern Medical University, Guangzhou, China; 2 State Key Laboratory of Oncology in South China, Guangdong Provincial Clinical Research Center for Cancer, Sun Yat-sen University Cancer Center, Guangzhou, Guangdong, China; 3 Department of Oncology, The First Affiliated Hospital, Sun Yat-sen University, Guangzhou, Guangdong Province China; 4 Department of Colorectal Surgery, The Sixth Affiliated Hospital, Sun Yat-Sen University, Guangzhou, China; 5 Cancer and Stem Cell Biology Program, Duke-NUS Medical School, Singapore, Singapore; 6 Laboratory of Cancer Epigenome, Division of Medical Sciences, National Cancer Centre, Singapore, Singapore; 7 Hainan Academy of Medical Science, Hainan Medical University, Haikou, China

**Keywords:** Cancer therapy, Prognostic markers

## Abstract

Concurrent chemotherapy is the standard treatment strategy for advanced-stage nasopharyngeal carcinoma (NPC). However, chemoresistance inevitable develops and the underlying mechanism remains poorly understood. In this study, we identify the arginine methyltransferase PRMT5 as a key gene associated with chemoresistance to paclitaxel in NPC. We demonstrate that PRMT5 facilitated paclitaxel resistance by inducing KCNMB4 expression in nasopharyngeal carcinoma cells. Mechanistically, PRMT5 is recruited to the promoter region of KCNMB4, where it catalyzes H3R2me2s and enhances KCNMB4 expression. Furthermore, elevated levels of PRMT5 or KCNMB4 correlated with poorer survival and higher recurrence rates in NPC patients. Notably, genetic or pharmacological inhibition of PRMT5 significantly sensitized NPC cells to paclitaxel, both in vitro and in vivo. Collectively, these results suggest that the PRMT5-KCNMB4 axis plays a crucial role in mediating chemoresistance in NPC and targeting this axis may provide a promising therapeutic strategy for late-stage NPC patients.

## Introduction

Nasopharyngeal carcinoma (NPC), arising from nasopharynx epithelium, is one of the most prevalent cancers in Southeast Asia and southern China [1]. As a highly radio- and chemo-sensitive cancer, early stage patients exhibit a favorable survival rate (85–90% for 5-year) [2]. However, chemoresistance invariably emerges, around 10% of patient experience recurrence or distant metastasis, leading to high mortality [3, 4]. Therefore, it is crucial to elucidate the underlying mechanism of chemoresistance and identify potential therapeutic target for advanced stage NPC patients.

Arginine methylation is a post-translational modification that is widely present in mammalian cells [5, 6]. It is as ubiquitous as phosphorylation and ubiquitination and regulates various biological processes, including transcription, cellular signaling, mRNA translation, DNA damage, receptor transport, protein stability, and pre mRNA splicing [7–10]. There are three types of arginine methyltransferases; type I enzymes (PRMT1, PRMT2, PRMT3, PRMT4, PRMT6 and PRMT8) generate asymmetric dimethylated species of target arginine; type II enzymes (PRMT5 and PRMT9) produce symmetric dimethylated species; and type III enzymes (PRMT7) catalyze the formation of momomethylation of arginine. PRMT5, the most extensively studied type II arginine methyltransferase, is generally considered as an epigenetic repressor. It represses gene expression through symmetric dimethylation of histones H4R3 (H4R3me2s), H3R8 (H3R8me2s) and H2AR3 (H2R3me2s) [11–13], while it can also activate gene transcription through demethylation of H3R2 (H3R2me2s) [14]. Abnormal expression of PRMT5 has been shown to contribute to drug resistance in cancer. In melanoma, inhibition of PRMT5 sensitizes melanoma cells to CDK4/6 inhibitor [15]. In breast cancer, PRMT5 modulates the sensitivity of breast cancer cells to doxorubicin by regulating OCT4/A, KLF4 and C-MYC [16]. Even though PRMT5 has been shown to promote the tumor progression and radioresistance of NPC [17], the role of PRMT5 in chemo-resistance in NPC has yet to be determined.

The *KCNMB4* (Potassium Calcium-Activated Channel Subfamily M Regulatory Beta Subunit 4) gene is located at 12q15 and encodes the modulatory transmembrane β subunits of the large conductance Ca^2+^-activated K^+^ channel (BK channel) [18, 19]. A growing number of studies highlights the important role of ion channels in oncogenesis [20, 21]. BK channels have been found to be overexpressed in various cancers, including astrocytoma, glioma, breast cancer, ovarian cancer and prostate cancer [22–26], where they contribute to tumor cell proliferation, migration and invasion. Additionally, BK channels regulate cell differentiation and contribute to the stemness of cancer stem cell [27]. Moreover, BK channel subunits have been implicated in promoting breast cancer development and modulating responses to endocrine therapy in preclinical models [28]. KCNMB4 regulates drug resistance. In the brain, KCNMB4 is highly expressed and renders the BK channel α subunit resistance to charybdotoxin and iberiotoxin [29]. In glioblastoma, blockage of BK channels inhibits hypoxia-induced migration and chemoresistance to cisplatin [30]. However, the potential role of BK channels in the chemoresistance of NPC has not yet been explored.

In the present study, through epigenetic compound library screening, we identified that PRMT5 inhibitors could overcome paclitaxel resistance in NPC. We demonstrated, for the first time, that PRMT5-KCNMB4 axis promotes chemoresistance of NPC and poor prognosis of NPC patients. Mechanistically, PRMT5 is recruited to the promoter region of KCNMB4, where it facilitates H3R2me2s and enhances gene expression of *KCNMB4*. Inhibition of PRMT5 significantly sensitized NPC cells to paclitaxel, both in vitro and in vivo. These findings suggested that drug screening is an effective approach to identify novel combinatorial drug targets and that PRMT5 inhibition is a promising way to overcome chemoresistance in NPC.

## Results

### Combinatorial epigenetic drug screening identifies PRMT5 inhibitors as sensitizers to paclitaxel

**Fig. 1:**
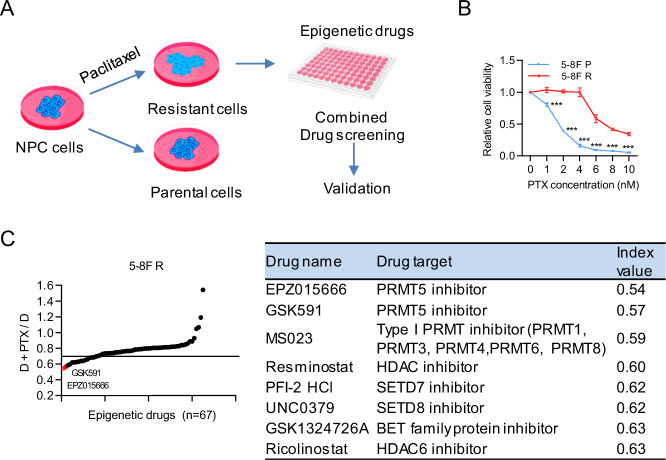
Combinatorial epigenetic drug screening identifies PRMT5 inhibitors as sensitizers to paclitaxel. **A** Outline of combinatorial drug screening. 5-8 F resistant (5-8 F R) cells were seeded in 96-well plate for 24 h before being treated with 67 epigenetic drugs (1.0 μmol/L) with or without paclitaxel ((2 nmol/L). **B** Paclitaxel cytotoxicity assay of 5-8 F parental (5-8 F P) and 5-8 F R cells. Cells were treated with different concentrations of PTX for 96 h. Data are shown as means ± SD (*n* = 3). *P < 0.05; **P < 0.01; ***P < 0.001. **C** Left, the combinatorial effects of 67 epigenetic drugs in 5-8 F R. An inhibition value (I) was defined for each drug. I = (Cell viability of combined treatment relative to the control) / (Cell viability of single drug treatment relative to the control). The black line indicates I = 0.7. A drug with an inhibitory value less than 0.7 was considered as a sensitizer of paclitaxel. The I value was determined according to the sensitivity of 5-8 F R to paclitaxel during the drug screening. It was adjusted to identify more effective drug candidates (lower than I value). **D** the cell viability of single drug treatment to the control. D + PTX, the cell viability of combined treatment relative to the control. Right, the top 8 hits of the drug screening; drugs were ranked by their I value.

To uncover potential new epigenetic drug targets to overcome chemoresistance in NPC, we performed a combinatorial epigenetic drug screen with paclitaxel using the previously established chemoresistance NPC cell line 5–8 F R (Fig. 1A) [31]. Cytotoxicity assays with paclitaxel confirmed that 5-8 F R cells exhibited increased resistance to paclitaxel (PTX) compared to parental cell line (Fig. 1B). 67 epigenetic drugs were tested (Supplementary Table S3). Drug candidates were ranked by their calculated value index, with values below 0.7 were considered as sensitizers (Fig. 1C). Sixteen epigenetic drugs demonstrated combinatorial effect, and the top eight candidates are presented. Notably, the top two candidates were PRMT5 inhibitors, which led us to select PRMT5 inhibitors for further study.

### PRMT5 inhibitors restore chemo-sensitivity of NPC cells

**Fig. 2:**
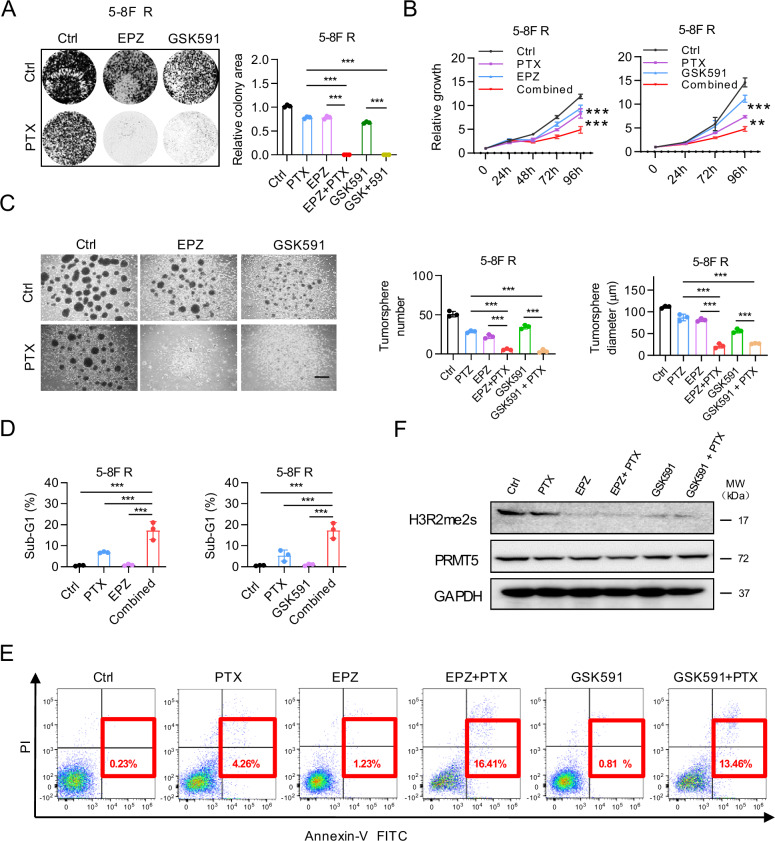
PRMT5 inhibitors restore chemo-sensitivity of NPC cells. **A** the effect of combined PRMT5 inhibitor and PTX treatment on cell proliferation, as shown by colony formation assay. The PTX concentration used was 2 nmol/L, and the concentration of EPZ015666 (EPZ) and GSK591 concentration were 3 μmol/L. The same concentrations of PRMT5 inhibitors and PTX were used in the other experiments, unless otherwise stated. Left, representative images; Right, quantification. Data are shown as means ± SD (*n* = 3). ***P < 0.001.**B** the growth curve of 5-8FR cells treated with PRMT5 inhibitors, with or without PTX, at different time points. Cell viability was assessed using CellTiter-Glo reagent. Data are shown as means ± SD (*n* = 3). *P < 0.05; **P < 0.01; ***P < 0.001. **C** Tumorsphere formation assay of 5-8 F R cells under different treatments for 8 days. Scale bars, 200 μm. Representative images (Left) and quantifications (Right). Data are shown as means ± SD (*n* = 3). ***P < 0.001. **D** Sub-G1 population analysis in 5-8 F R cells treated with PRMT5 inhibitors, PTX or both for 96 h. Data are shown as means ± SD (*n* = 3). *P < 0.05; **P < 0.01; ***P < 0.001. **E** Annexin-V-PI staining to assess the apoptotic ratios of 5–8 F R cells under different treatments. Cells were collected after 96 h of treatment. Results shown are representative images from three experiments. **F** Western blot analysis of PRMT5 and H3R2me2s expression in 5-8 F R cells under different treatment. EPZ015666 (EPZ).

To further validate the combinatorial effects of PRMT5 inhibitors (PRMT5i) and paclitaxel in chemoresistant NPC, we conducted colony formation assay (Fig. 2A) and time-course cell proliferation assay (Fig. 2B) in 5-8 F R cells. The combinatorial treatments significantly reduced cell proliferation, whether using EPZ015666 (EPZ) or GSK591. Tumorsphere formation in 5-8 F R cells was nearly completely inhibited by the combinatorial treatments, indicating remarkable restoration of chemosensitivity (Fig. 2C). Moreover, the combination dramatically increased cell death and apoptosis of 5-8 F R cells (Fig.2D, E, Supplementary Fig. 1A). Similar results were observed in other NPC cell lines (Supplementary Fig. S1B–D). Consistent with their mechanism of action, pharmacological inhibition of PRMT5 did not affect total PRMT5 protein levels, but significantly reduced its methyltransferase activity, as evidenced by decreased H3R2me2s levels (Fig. 2F). Intriguingly, we found that PRMT5 inhibitor could also enhance the antitumor effect of other chemotherapies in NPC (Supplementary Fig. S1E), significantly expanding its potential clinical utility. Altogether, these results imply that PRMT5 inhibitors enhance the sensitivity of NPC cells to paclitaxel by retarding cell proliferation and inducing apoptosis.

### PRMT5 confers chemo-resistance of NPC and associates with poor prognosis of NPC patients

**Fig. 3:**
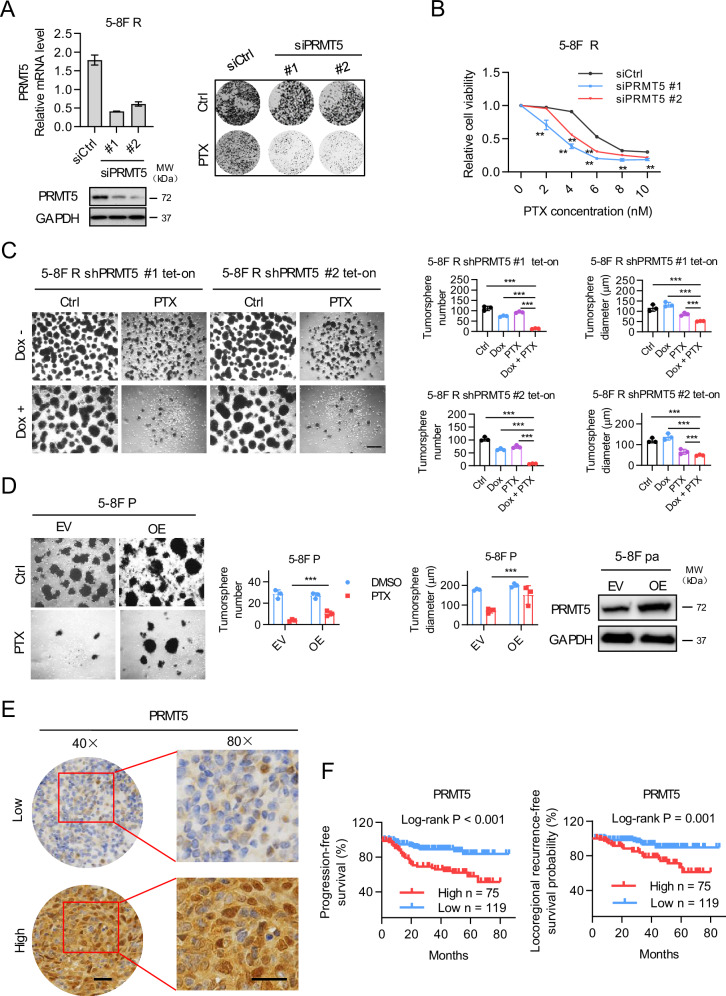
PRMT5 confers chemo-resistance of NPC and associates with poor prognosis of NPC. **A** Right, PRMT5 expression as shown by qRT-PCR and western blot analysis in siRNA treated 5-8 F R cells. Left, the effects of PRMT5 downregulation on cell sensitivity to PTX as shown by colony formation assay. **B** Cell cytotoxicity assay of PTX. Cells were treated with different concentrations of PTX for 96 h. Cell viability was assessed using CellTiter-Glo reagent. Data are shown as means ± SD (*n* = 3). *P < 0.05; **P < 0.01. **C** Tumorsphere formation assay of shPRMT5 Tet-on 5-8 F R cells treated with Dox, with or without PTX. Representative images (left) and quantifications (right). Bars represent the means ± SD (*n* = 3). Scale bars, 200 μm. *P < 0.05; **P < 0.01; ***P < 0.001. **D** Tumorsphere formation assay of 5–8 F P cells with PRMT5 overexpression with or without PTX treatment. Representative images (Left) and quantifications (Right). Bars represent the means ± SD (*n* = 3). Scar bars, 400 μm. *P < 0.05; **P < 0.01; ***P < 0.001. **E** IHC analysis of PRMT5 expression in 194 nasopharyngeal carcinoma samples from patients treated with chemotherapy. PRMT5 was detected in both the nucleus and cytosol. IHC scores were calculated by multiplying the scores for the proportion of positively stained tumor cells (1, <10%; 2, 0%–50%; 3, 50%–80%; 4, >80%) and staining intensity (0, no staining; 1, weak; 2, moderate; 3, strong) by each investigator, then averaged. IHC score > 8 was used to classify tumors with high PRMT5 expression and the rest were defined as low expression. Representative images are shown. Scale bar 10 μm. **F** The Kaplan–Meier survival analysis for progression-free survival and locoregional recurrence-free survival of 194 patients with nasopharyngeal carcinoma with different PRMT5 expression levels, as determined in D.

To determine the role of PRMT5 in paclitaxel resistance of NPC, we first assessed the effect of PRMT5 knockdown on the response of 5-8 F cells to PTX treatment. PRMT5 downregulation by two independent siRNAs in 5-8 F R cells enhanced PTX-mediated growth suppression, as demonstrated by colony formation assays (Fig. 3A). Cytotoxicity assays revealed that PRMT5 depletion significantly sensitized 5-8 F R cells to PTX treatment (Fig. 3B). Additionally, an inducible PRMT5 knockdown system was established in 5-8 F R cells. Conditional knockdown of PMRT5 by doxycycline (Dox) enhanced the PTX-induced suppression of tumorsphere formation (Fig. 3C and Supplementary Fig. S2A). Conversely, overexpression of PRMT5 in 5-8 F P cells enhanced the tumorsphere formation in response to paclitaxel (Fig. 3D). Furthermore, PRMT5 expression was found to be upregulated in 5-8 F R cells compared to 5-8 F P cells (Supplementary Fig. S2B). To assess the clinical relevance of PRMT5, immunohistochemistry (IHC) was performed on 194 nasopharyngeal carcinoma samples from chemotherapy-treated patients (Supplementary Tables S4 and S5). PRMT5 was detected both in nucleus and cytoplasm (Fig. 3E). Kaplan-Meier survival analysis revealed that high PRMT5 expression was negatively associated with progression-free survival and locoregional recurrence-free survival in NPC patients (Fig. 3F). Collectively, these data suggest that PRMT5 overexpression renders NPC cells resistant to PTX and is associated with poor prognosis of NPC patients.

### PRMT5 epigenetically regulates KCNMB4 expression

**Fig. 4:**
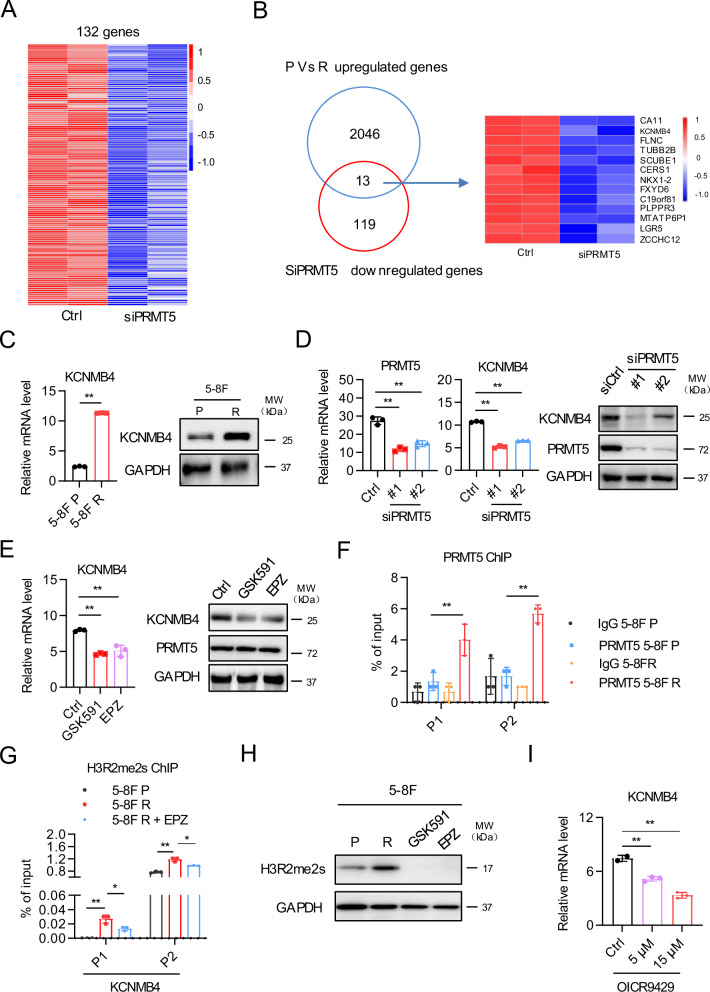
PRMT5 epigenetically regulates KCNMB4 expression. **A** Heatmap for differentially expressed genes in PRMT5 knockdown and control 5-8 F R cells. Downregulation genes were shown after depletion of PRMT5**. B** Venn diagram showing the overlapping genes obtained from upregulated genes of 5-8 F R vs. 5-8 F P and A. **C** qRT-PCR and western analysis of KCNMB4 expression in 5-8 F P and 5-8 F R cells. Data are shown as means ± SD (*n* = 3). *P < 0.05; **P < 0.01. **D** qRT-PCR and western analysis of PRMT5 and KCNMB4 expression in siPRMT5-treated 5-8 F R cells. Data are shown as means ± SD (*n* = 3). *P < 0.05; **P < 0.01. **E** qRT-PCR and western analysis of KCNMB4 expression in 5-8 F R cells treated with PRMT5 inhibitors. EPZ015666 (EPZ). Data are shown as means ± SD (*n* = 3). *P < 0.05; **P < 0.01. **F** Enrichment of PRMT5 or rabbit IgG at the promoter region of KCNMB4. Data are shown as means ± SD (*n* = 3). *P < 0.05; **P < 0.01. **G** H3R2me2s enrichment at the KCNMB4 promoter in 5-8 F P, 5-8 F R and 5-8 F R cells with EPZ treatment. Data are shown as means ± SD (*n* = 3). *P < 0.05; **P < 0.01. **H** Western blot results showing the expression of H3R2me2s of 5-8 F P, 5-8 F R and 5-8 F R cells with EPZ treatment. **I** qRT-PCR results showing the expression of KCNMB4 in 5-8 F R cells with different concentrations of WDR5 inhibitor (OICR9429) treatment. Data are shown as means ± SD (*n* = 3). *P < 0.05; **P < 0.01.

To identify downstream target of PRMT5 that may mediate paclitaxel resistance in NPC, we performed RNA sequencing of 5-8 F R cells treated with PRMT5 siRNA. RNA sequencing data of 5-8 F P and 5-8 F R cells from our previous study was utilized for data mining [31]. We focused on genes that were overexpressed in 5-8 F R cells compared with 5-8 F P, while being downregulated upon PRMT5 knockdown. A total of 2059 genes upregulated in 5-8 F R cells (Supplementary Fig. S3A), and 132 genes were downregulated by PRMT5 depletion (Fig. 4A). Mapping these two clusters of genes, 13 genes were found in common (Fig. 4B). Since BK channels have been implicated in chemoresistance previously [30], we selected KCNMB4 for further investigation. As expected, KCNMB4 was overexpressed in 5-8 F R cells at both mRNA and protein levels, compared with 5-8 F P cells (Fig. 4C). Furthermore, PRMT5 knockdown reduced KCNMB4 expression in 5-8 F R (Fig. 4D). Notably, treatments with PRMT5 inhibitors also led to downregulated of KCNMB4 expression (Fig. 4E).

To determine whether PRMT5 regulates KCNMB4 expression directly, we performed PRMT5 chromatin immunoprecipitation (ChIP). ChIP-qPCR results demonstrated that PRMT5 binds to the promoter region of KCNMB4, with significantly more PRMT5 protein recruited to the promoter in 5-8 F R cells compared to 5-8 F P cells (Fig. 4F). PRMT5 has been reported to activate gene expression via H3R2 methylation (H3R2me2s), which is recognized by WDR5 to promote transcription [32]. Our ChIP-qPCR results verified that H3R2me2s were more enriched at the KCNMB4 promoter in 5-8 F R cells than in 5-8 F P cells, and PRMT5 inhibitor treatment reduced H3R2me2s levels at the KCNMB4 promoter (Fig. 4G). Western blot results further confirmed this finding (Fig. 4H). Moreover, WDR5 inhibition downregulated KCNMB4 expression (Fig. 4I) and sensitized 5-8 F R cells to PTX (Supplementary Fig. S3B). Together, these results imply that PRMT5 regulates KCNMB4 expression through H3R2me2s-mediated transcription activation.

### KCNMB4 confers resistance to paclitaxel and is correlated with poor prognosis in nasopharyngeal carcinoma

**Fig. 5:**
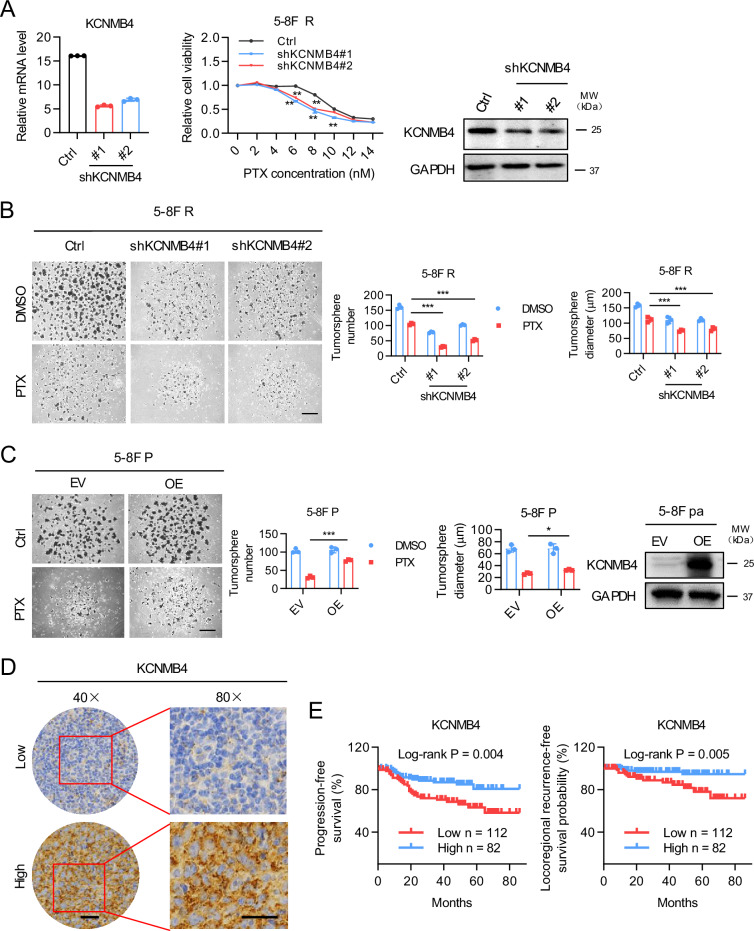
KCNMB4 confers resistance to paclitaxel and is correlated with poor prognosis in nasopharyngeal carcinoma. **A** KCNMB4 expression as shown by qRT-PCR (Left) and western blot (Right) analysis in stable knockdown KCNMB4 5-8 F R cells. Middle, the effects of PRMT5 downregulation on cell sensitivity to PTX as shown by proliferation assay. Data are shown as means ± SD (*n* = 3). *P < 0.05; **P < 0.01. **B** Tumorsphere formation assay of stable KCNMB4 knockdown 5-8 F R cells under PTX treatment. Representative images (Left) and quantifications (Right). Bars represent the means ± SD (*n* = 3). Scale bars, 200 μm. *P < 0.05; **P < 0.01; ***P < 0.001. **C** Tumorsphere formation assay of 5-8 F R cells with KCNMB4 overexpression (OE) in the presence or absence of PTX. Representative images (Left) and quantifications (Middle). Bars represent the means ± SD (*n* = 3). Scale bars, 200 μm. *P < 0.05; **P < 0.01; ***P < 0.001. Right, PRMT5 expression as shown by western blot analysis. **D** IHC analysis of KCNMB4 expression in 194 nasopharyngeal carcinoma samples from patients treated with chemotherapy. KCNMB4 was detected on membrane as it is a membrane protein. IHC scores were calculated by multiplying the scores for the proportion of positively-stained tumor cells (1, <10%; 2, 0–50%; 3, 50–80%; 4, >80%) and staining intensity (0, no staining; 1, weak; 2, moderate; 3, strong) by each investigator, then averaged. IHC score > 6 was used to classify tumors with high KCNMB4 expression and the rest was defined as low expression. Representative images are shown. Scale bar 10 μm. **E** The Kaplan–Meier survival analysis for progression-free survival and locoregional recurrence-free survival of 194 patients with nasopharyngeal carcinoma with different KCNMB4 expression levels as determined in **D**.

We next investigated the role of KCNMB4 in the chemoresistance of NPC. Downregulation of KCNMB4 significantly sensitized 5-8 F R cells to paclitaxel treatment, as evidenced by proliferation assay (Fig. 5A). Stable knockdown of KCNMB4 also reduced the tumorsphere formation ability of 5-8 F R cells under paclitaxel treatment, suggesting a loss of cancer stemness (Fig. 5B). Conversely, overexpression of KCNMB4 in 5-8 F P cells enhanced the tumorsphere formation and proliferation in response to paclitaxel (Fig. 5C, Supplementary Fig. S4A). Notably, KCNMB4 overexpression significantly attenuated the chemosensitization effect of PRMT5 depletion in 5-8 F R cells (Fig. S4B). To assess the clinical relevance of KCNMB4, immunohistochemistry (IHC) analysis was performed on 194 nasopharyngeal carcinoma patient samples treated with chemotherapy (Supplementary Tables S6 and S7). KCNMB4 was detected on the membrane, consistent with its role as a membrane protein (Fig. 5D). The Kaplan-Meier survival analysis demonstrated that high expression of KCNMB4 was negatively correlated with progression-free survival and locoregional recurrence-free survival of NPC patients (Fig. 5E). Furthermore, Pearson correlation analysis revealed a positive correlation between PRMT5 and KCNMB4 protein expression levels in our clinical cohort (Supplementary Fig. S4C). All together, these results indicate that elevated expression of KCNMB4 contributes to chemoresistance in NPC and is associated with poor prognosis in NPC patients.

### PRMT5 downregulation restores the chemo-sensitivity of NPC in vivo

**Fig. 6:**
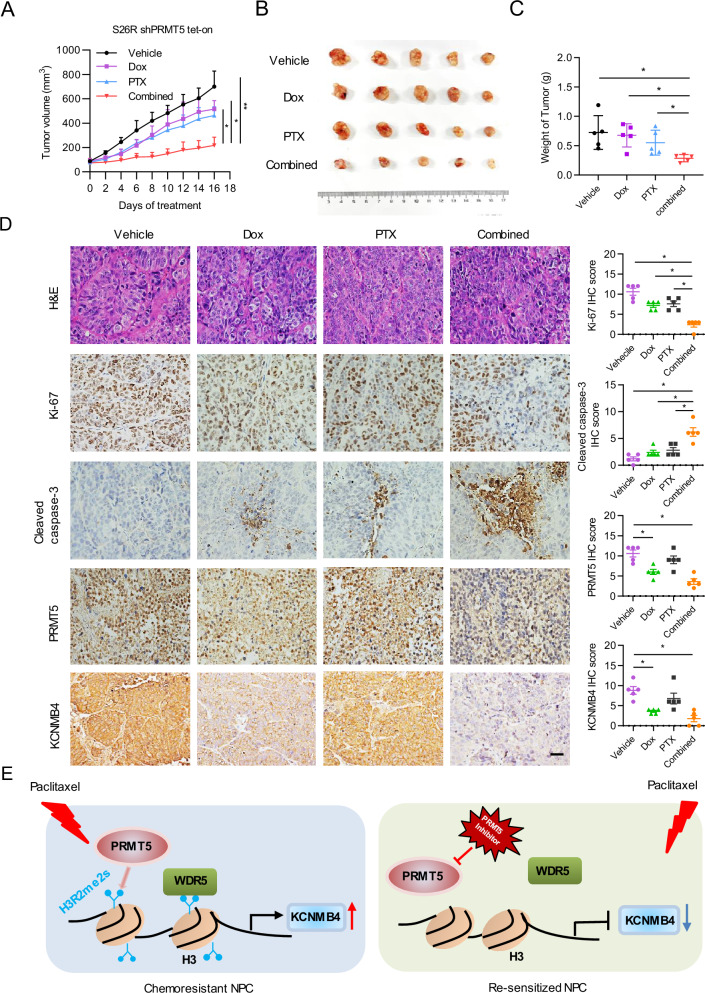
PRMT5 downregulation restores the chemo-sensitivity of NPC in vivo. **A** Xenograft tumor growth curve of S26 R shPRMT5 Tet-on cells under different treatments. Mice were treated with doxycycline (Dox) at 75 mg/kg, paclitaxel (PTX) at 5 mg/kg, or both for 16 days. Error bars represent mean ± SEM (*n* = 5 per group). *P < 0.05; **P < 0.01; ***P < 0.001 (independent t test). **B** Images show extracted tumors at the end of the experiments. **C** Bar graph shows the tumor weight at the end of the treatment. Data are presented as mean ± SD. *P < 0.05; **P < 0.01; ***P < 0.001 (independent t test). **D** Left, representative images of HE, KI-67, cleaved caspase-3 and PRMT5 IHC staining of tumor sections from mice. Right, IHC-scores of KI-67, cleaved caspase-3, and PRMT5 in tumor sections with different treatments. IHC scores were calculated by multiplying the scores for the proportion of positively-stained tumor cells (1, <10%; 2, 0–50%; 3, 50–80%; 4, >80%) and staining intensity (0, no staining; 1, weak; 2, moderate; 3, strong) by each investigator, then averaged. Scale bar 20 μm. P values were calculated with two-tailed *t* test. *P < 0.05; **P < 0.01. **E** Schematic representation illustrating the involvement of the PRMT5-KCNMB4 axis in promoting paclitaxel resistance in NPC.

Finally, we investigated to see whether inhibition of PRMT5 expression could restore the chemosensitivity of NPC tumors to PTX treatment. Due to the low tumorigenic potential of 5-8 F cells, we utilized the S26 R NPC cell line from a previous study [33]. In vitro experiments demonstrated that PRMT5 knockdown significantly sensitized S26R cell to PTX treatment (Supplementary Fig. S5A–C). For in vivo validation, PRMT5 inducible knockdown S26 R cells were subcutaneously engrafted into nude mice. The mice were randomly divided into four groups: one group received vehicle, while the other three groups received doxycycline (Dox), paclitaxel (PTX) alone, or combined Dox and PTX treatment. Treatment with either Dox or PTX alone delayed tumor growth; however, the combined treatment almost arrested tumor growth with a minimal body weight loss (Fig. 6A–C, Supplementary Fig. S5D–H). Immunohistochemistry (IHC) analysis confirmed significant downregulation of PRMT5 and KCNMB4 expression in tumors treated with DOX or combined treatment (Fig. 6D), validating target inhibition. Furthermore, the combined treatment significantly reduced the number of Ki-67 positive cells and increased the cleaved caspase-3 expression in tumors compared to the control or individual drug treatment. Taken together, these data demonstrate that downregulation of PRMT5 restores the chemo-sensitivity of NPC tumors to paclitaxel.

## Discussion

Docetaxel-cisplatin-5-fluorouracil (TFP) is an effective induction chemotherapy for advanced-stage NPC patients [34], however, a subset of patients develops chemoresistance and distant metastasis, leading to treatment failure. Therefore, it is crucial to elucidate the underlying mechanisms driving this resistance. In recent years, an increasing number of studies have demonstrated that PRMT5 inhibitors, already used in clinical trials [35, 36] can sensitize cancer cells to various therapeutics. For instance, the specific PRMT5 inhibitor EPZ015666 has been shown to overcome resistance to mTOR inhibitors in glioblastoma [37]. In pancreatic cancer, PRMT5 is identified as a synthetic lethality target in combination with gemcitabine [38]. In lung cancer, EPZ015666 impairs radioresistance [39], while in breast cancer, inhibiting PRMT5 suppresses the growth of paclitaxel-resistant cancer cells [40]. Similarly, we found that PRMT5 inhibitors can overcome paclitaxel resistance in NPC. Thus, targeting PRMT5 represents a promising strategy to overcome chemoresistance in NPC.

H3R2me2s a histone mark that maintains genes in a poised state within euchromatin, ready for transcription activation. It recruits WDR5 to chromatin, where it forms protein complexes with other factors to initiate transcription [32]. PRMT5 catalyzes H3R2me2s, and studies have shown that PRMT5 can activate gene expression through this modification. For example, in breast cancer, PRMT5 upregulates FOXP1 expression via H3R2me2s [14]. Consistently, our study demonstrates that PRMT5 regulates KCNMB4 expression, which is elevated in NPC, by H3R2me2s. Furthermore, PRMT5 inhibitors, which retrain the enzyme activity of PRMT5, reduce KCNMB4 expression. Our further results provide evidence that the PRMT5-KCNMB4 axis plays a crucial role in the chemoresistance of NPC and correlates with inferior prognosis in NPC patients.

While our study elucidates the role of the PRMT5-KCNMB4 axis in NPC chemoresistance, we acknowledge that the result was primarily conducted in a single cell line. This limitation is partially mitigated by our clinical correlation data, which demonstrate that PRMT5 and KCNMB4 overexpression consistently associate with poor prognosis in a large patient cohort. Nevertheless, further in vitro and in vivo validation across diverse NPC cell models will be essential to confirm the broader applicability of PRMT5 inhibition as a therapeutic strategy. Moreover, the inhibitor targeting PRMT5 has already entered Phase II clinical trials for treating with relapsed/refractory hematologic malignancies [41], demonstrating its therapeutic potential.

In summary, our study uncovers that PRMT5 drives KCNMB4 expression through H3R2me2s by which PRMT5 induced chemoresistance and poor prognosis in NPC patients. Importantly, targeting PRMT5 significantly sensitized nasopharyngeal carcinoma cells to paclitaxel both in vitro and in vivo (Fig. 6E). These findings provide new insights into the multifaceted nature of chemotherapy resistance in NPC and suggest that PRMT5 may serve as a potential therapeutic target for improving treatment outcomes.

## Materials and methods

### Patient samples, cell culture and reagent

This work was approved by the Institutional Ethical Review Board of Guangdong Provincial People’s Hospital (S2024-281-01). Written informed consent was obtained from each patient who provided the tissue. Human nasopharyngeal carcinoma cell lines 5-8 F, S18, CNE2, 6-10B and HEK293T (RRID:CVCL_0063) cells were grown in RPMI-1640 (Invitrogen) or DMEM (Invitrogen) supplemented with 10% FBS and 1% penicillin-streptomycin (Gibco BRL) at 37°C in 5% CO2 incubator. All cell lines were tested negative for mycoplasma contamination. All cell lines had been authenticated and were generously provided by Dr. M. Zeng (Sun Yat-sen University Cancer Centre). Paclitaxel-resistant cell lines were generated by treating parental cells with escalating concentrations of paclitaxel for three months, until a certain concentration was achieved. Epigenetic inhibitor library used in drug screening was purchased from Selleck Chemicals. PRMT5 inhibitor EPZ015666 and GSK591 were purchased from TargetMol.

### Combinatorial drug screening

Resistant 5-8 F R cells were seeded in 96-well plates and treated with 67 epigenetic inhibitors, with or without paclitaxel, for 96 h in the drug screening. Cell viability was assessed by the CellTiter Glo reagent (Promega) according to the manufacturer’s instructions. An inhibitory value I was determined as follows: (The cell viability of combined treatment relative to the control) / (The cell viability of single inhibitor treatment relative to the control). I < 0.7 was considered as a potential sensitizer to paclitaxel. The I value was determined according to the sensitivity of 5-8 F R cells to paclitaxel and adjusted to identify more effective candidates.

### Colony formation, cell proliferation, tumorsphere formation

For colony formation assay, 1×10^4^ cells were seeded into 6-well plates for about 8 days with indicated drug treatment, and were stained with crystal violet. They were quantified using ImageJ/Fiji software(https://fiji.sc/). For cell proliferation assay, 2×10^3^ cells were seeded in 96-well plate and 24 h later, cells were treated with different compounds. Cell viability was tested every day for 4 days using CellTiter-Glo reagent. For the tumorsphere formation assay, a single cell suspension (1×104) was cultured in 6-well ultra-low-attachment plates with MammoCult Medium, supplemented with heparin (1:500) and fresh hydrocortisone (0.5 μg/mL). After 10–12 days, tumorspheres were stained with 2-(4-iodo-phenyl)-3-(4-nitrophenyl)-5-phenyl-2-Htetrazolium chloride (INT) (Sigma-Aldrich) and photographed. Images and quantification were done using Olympus CellSens Dimension software.

### Flow cytometry analysis

PI staining was used to quantify the sub-G1 population, which reflects the extent of cell death. Cells were treated with different compounds for 72 h, then fixed and stained with PI (50 μg/mL). The stained cells were analyzed using SP6800 Spectral Cell Analyzer and quantified with FlowJo software (RRID: SCR_008520). For the apoptosis assay, cells were treated with indicated drugs for 72 h, then labeled with Annexin V/PI according to the manufacturer’s instructions and immediately analyzed by flow cytometry.

### siRNA, shRNA and plasmid transfection

siRNAs for PRMT5, KCNMB4, were purchased from Genepharma (Shanghai, China). Transfection was performed using lipofectamine RNAiMax reagents (Thermo Fisher Scientific) according to the manufacturer’s instructions. The sequences of the siRNAs are shown in Supplementary Table S1.

To generate PRMT5 inducible knockdown cell lines, the Tet-pLKo-puro plasmid (RRID: Addgene_21915) was used. shRNA targeting PRMT5 was subcloned into Tet-pLKO-puro. shRNA for KCNMB4 was purchased from Genepharma (Shanghai, China). For KCNMB4 overexpression, cDNA of KCNMB4 was amplified and cloned into pLVX-AcGFP1-N1 plasmid (Novopro Bioscience Inc., V012707). Virus packaging was conducted by co-transfection of psPAX2 (RRID: Addgene_12260) and pMD2.G (RRID: Addgene_12260) along with viral vectors into 293 T. Virus supernatant was collected 48 h later. Target cells were transduced with virus supernatant and selected with GFP by FACS or puromycin (1.0 μg/mL).

### qRT-PCR

Total RNA was extracted using Rapid Total RNA Extraction Kit (Goonie) according to the manufacturer’s protocol. qRT-PCR was performed using PerfectStart Green qPCR SuperMix (TransGen Biotech) on a Bio-Rad PCR detection system. Expression of 18S was used as internal control form normalization. Primer sequences are listed in Supplementary Table S2.

### Immunoblotting

Total cell lysates were extracted from cell lines using RIPA buffer with protease inhibitors. Protein lysates were then separated by SDS-PAGE gel and transferred to a PVDF membrane. The primary antibodies used were: PRMT5 (Abcam Cat# ab109451, RRID: AB_10863428), GAPDH (Cell Signaling Technology Cat# 2118, RRID: AB_561053). HRP-conjugated secondary antibodies used were: anti-rabbit (Cytiva Cat# NA934, RRID: AB_772206) and anti-mouse (Cytiva Cat# NA931, RRID: AB_772210). Immunoblot bands were detected using the Bio-Rad ChemiDoc MP imaging system.

### RNA-seq

For siPRMT5 RNA sequencing, cells were seeded in 6-well plates one day before transfected with siRNAs. After 48 h, cells were collected. Total RNA was extracted using RNeasy Mini kit (Qiagen). RNA-seq libraries were prepared using TruSeq Stranded RNA HT Kit (Illumina, 15032620) according to the manufacturer’s protocol. Samples were sequenced on the Illumina Hiseq2500 platform with paired-end reads of 150 bases. All the clean reads were aligned to the reference human genome (GRCh38, hg38) with STAR (RRID:SCR_004463). Differentially expressed genes were called using DESeq2 R package (version:1.28.1, RRID: SCR_000154) with | log2 fold change | ≥ 1 and adjusted *p*-value < 0.05. RNA-seq data.

### Chromatin immunoprecipitation qPCR

For PRMT5 chromatin immunoprecipitation, 5×10^6^ 5-8 F P or 5-8 F R cells were crosslinked with 1% formaldehyde at RT and then quenched with 0.125 M glycine. Cell nuclei were isolated by washing with 0.1% SDS lysis buffer (50 mM HEPES-KOH pH7.5, 150 mM NaCl, 2 mM EDTA, 1% Triton X-100, 0.1% Sodium deoxycholate, 0.1% SDS) with protease inhibitor. Nuclei were lysed with 1% SDS lysis buffer with protease inhibitor and sonicated on ice. Chromatin extract was then precleared with protein G Dynabeads and incubated with specific antibody-binding magnetic beads (5 μg Rabbit IgG, 5 μg PRMT5 Abcam Cat# ab109451, RRID: AB_10863428) overnight at 4 °C. Immunoprecipitated DNA was eluted, reverse cross-linked and purified for ChIP qPCR.

For H3R2me2s chromatin immunoprecipitation, 1×10^6^ 5-8 F P, 5-8 F R or 5-8 F R with EPZ-treated cells were crosslinked with 1% formaldehyde at RT and then quenched with 0.125 M glycine. The cells were lysed with 1% SDS lysis buffer and sonicated on ice. Chromatin was precleared with protein G Dynabeads and incubated with antibody-beads (H3R2me2s, Epigentek Cat# A-3705, RRID:AB_3668639) overnight at 4 °C. Immunoprecipitated DNA was eluted, reverse cross-linked and purified for Chip qPCR. Primers were designed around the promoter region of KCNMB4, sequences are listed in Supplementary Table S2.

### Xenograft studies

Six-week-old female BALB/c mice (RRID: MGI:2683685) were purchased from Beijing Vital River Laboratory Animal Technology Company. Mice were kept within animal room limits of 20-23°C and 40-60% humidity. All animal care and experimental procedures were approved by the Ethics of Animal Experiments of Guangdong People’s Hospital. For PRMT5-inducible knockdown xenograft experiment, mice were implanted subcutaneously in the flank with 6.5 × 10^5^ S26 R cells with inducible shPRMT5. Tumor volume was calculated using the formula 0.5×L×W^2^, where L and W are tumor length and width respectively. When tumors volume reached 100 mm^3^, mice were randomly divided into four groups: vehicle-treated, paclitaxel-treated, doxycycline-treated and combined treatment. Paclitaxel was administered via intraperitoneal injection every other day (5 mg/kg). Doxycycline was given by oral gavage at 75 mg/kg every day. When the tumor sizes of the vehicle group exceeded 1000 mm^3^, mice were euthanized, and tumors were collected for further analysis. No statistical method was used to predetermine the sample size for this experiment. No data was excluded and the investigator was blinded to the group allocation during the experiment.

### Immunohistochemistry staining

Ki-67, Caspase-3, PRMT5 and KCNMB4 protein expression were determined on formalin-fixed, paraffin-embedded sections by immunohistochemistry (IHC) staining. The procedure of IHC was performed as previously published [33]. After deparaffiniztion, the sections were subjected to antigen retrieval and probed with the following primary antibodies: PRMT5 (Abcam Cat#ab109451, RRID: AB_10863428), KCNMB4 (Proteintech Cat# 60122-1-Ig, RRID: AB_2280832), Ki-67 (ZSGB-Bio Cat#ZM-0167, RRID: AB_2920617) and cleaved caspase-3 (Cell Signaling Technology Cat# 9661, RRID: AB_2341188). IHC scores were determined by two experts blinded to treatment.

### Statistics

Statistical analysis was performed using Graphpad Prism version 7.0 for Windows. All experiments were repeated in triplicate, and data are given as mean ± SD, unless otherwise stated. The two-tailed Student t-test was used for comparing two independent groups unless otherwise stated. *P* < 0.05 was considered as the significance threshold.

## Supplementary information

Supplementary Figures S1-S5

Supplementary Tables S1-S7

Supplementary uncut Western blot

Supplementary raw data

AJ Checklist

## Data Availability

The raw data generated in this study are provided in the Supplementary Information files (Supplementary raw data and Supplementary uncut western blot). Publicly available RNA-seq datasets analyzed during this work are accessible through NCBI GEO (GSE214025). Additional supporting data are available from the corresponding author upon reasonable request.
