# Supplementary material for: PRMT5 upregulates KCNMB4 expression via histone methylation to promote paclitaxel resistance in advanced nasopharyngeal carcinoma
Source: Cell Death Dis. 2026 Jan 9;17(1):19. doi: 10.1038/s41419-025-08190-y (PMC12789566; doi:10.1038/s41419-025-08190-y)
Supplement: Supplementary file 1 — Supplementary Figures S1-S5 [file 41419_2025_8190_MOESM1_ESM.pdf]

Supplementary Figure 1

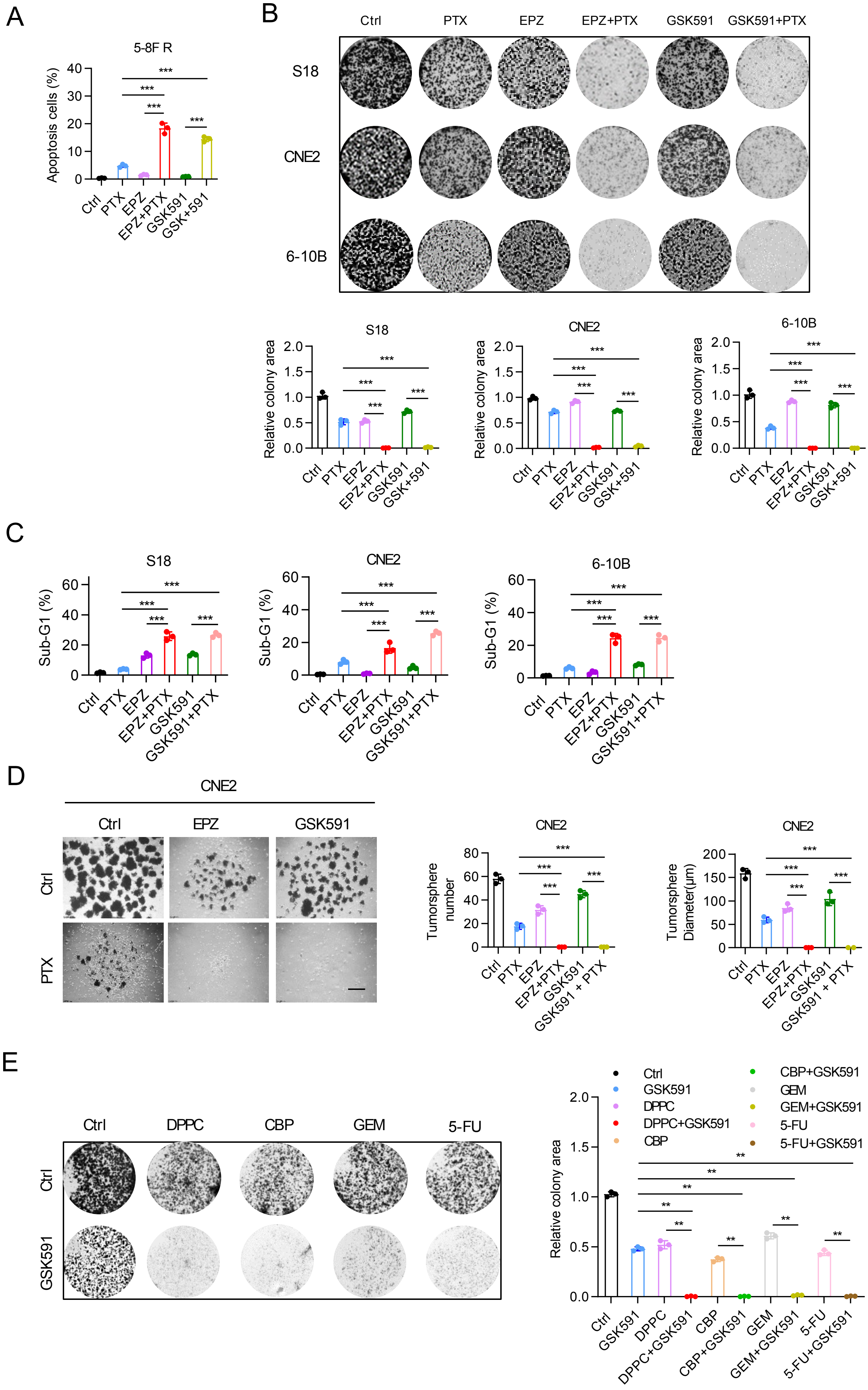

Supplementary Figure 1. PRMT5 inhibitors restore chemo-sensitivity of NPC cells. A, Quantification results of Annexin-V-PI staining in Figure 2E. Data are shown as means ± SD (n = 3). ***P < 0.001. **B,** the effect of combined PRMT5 inhibitor and PTX treatment on cell proliferation as shown by colony formation assay. EPZ015666 (EPZ) and GSK591 concentration used were 3 μmol/L. PTX concentration was optimized for each cell line. Up, representative images; Down, quantification. Data are shown as means ± SD (n = 3). ***P < 0.001. **C,** Sub-G1 population analysis in different cell lines treated with PRMT5 inhibitors, PTX or both for 96 h. Data are shown as means ± SD (n = 3). *P < 0.05; **P < 0.01; ***P < 0.001.**D,** Tumorsphere formation assay of CNE2 cells under PRMT5 inhibitor treatment with or without paclitaxel for 8 days. Representative images (left) and quantifications (right). Bars represent the means ± SD (n = 3). *, P < 0.05; **, P < 0.01; ***, P < 0.001. Scale bars, 200 μm. **E,** Colony formation assay of 5-8F R cells under different treatment. CBP, carboplatin; CDDP, cisplatin; GEM, gemcitabine; 5-FU, Fluorouracil. Left, representative images; Right, quantification. Data are shown as means ± SD (n = 3). **P < 0.001.

Supplementary Figure 2

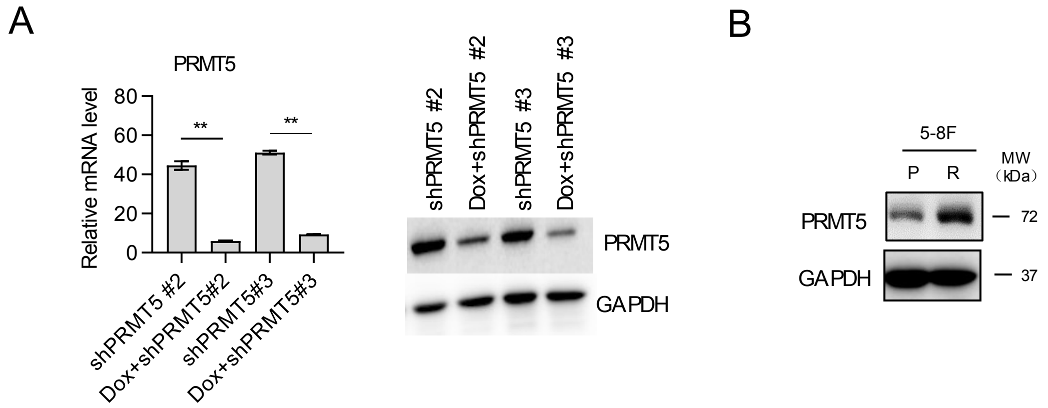

Supplementary Figure 2. Inducible knockdown of PRMT5 and its expression profiles were assessed in paired cell lines. A, PRMT5 expression as shown by qRT-PCR and western blot analysis with or without Dox treatment. Data are shown as means ± SD (n = 3). $^{*}P < 0.05$; $^{**}P < 0.01$. **B,** Western blot analysis of PRMT5 in 5-8F P and 5-8F R cells. Results shown are representative images from three experiments.

Supplementary Figure 3

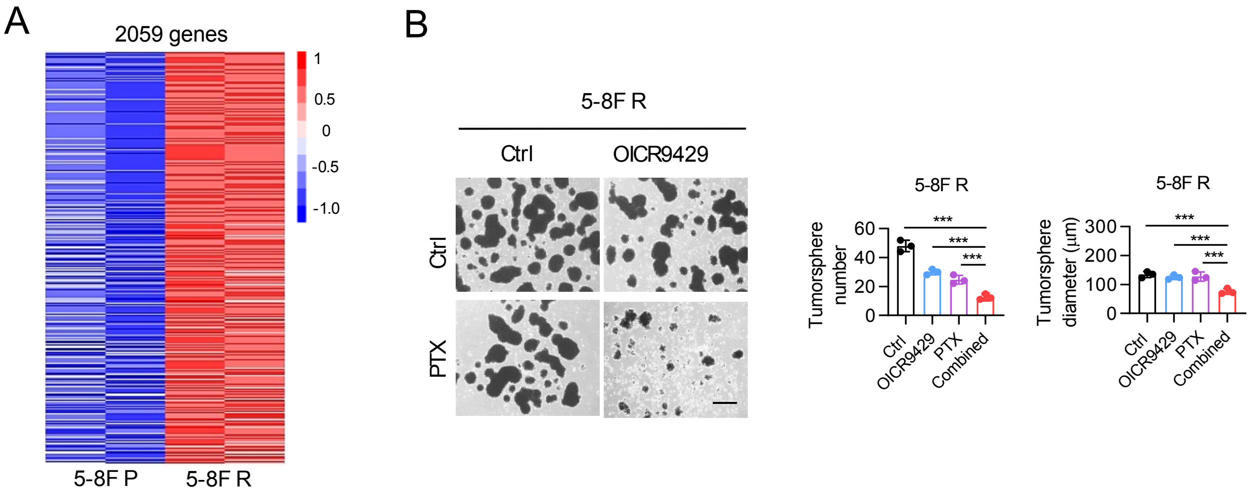

Supplementary Figure 3. RNA-seq results for 5-8F P and R cells, along with the effects of WDR5 inhibitor treatment on 5-8F R cells. A, Heatmap showing differentially expressed genes between 5-8F P and 5-8F R cells. Upregulated genes are shown (5-8F R vs 5-8F P). **B,** Tumorsphere formation assay of 5-8F R cells treated with WDR5 inhibitor (OICR9429). Representative images (Left) and quantifications (Right). Bars represent the means ± SD (n = 3). Scale bars, 200 μm. *P < 0.05; **P < 0.01; ***P < 0.001.

Supplementary Figure 4

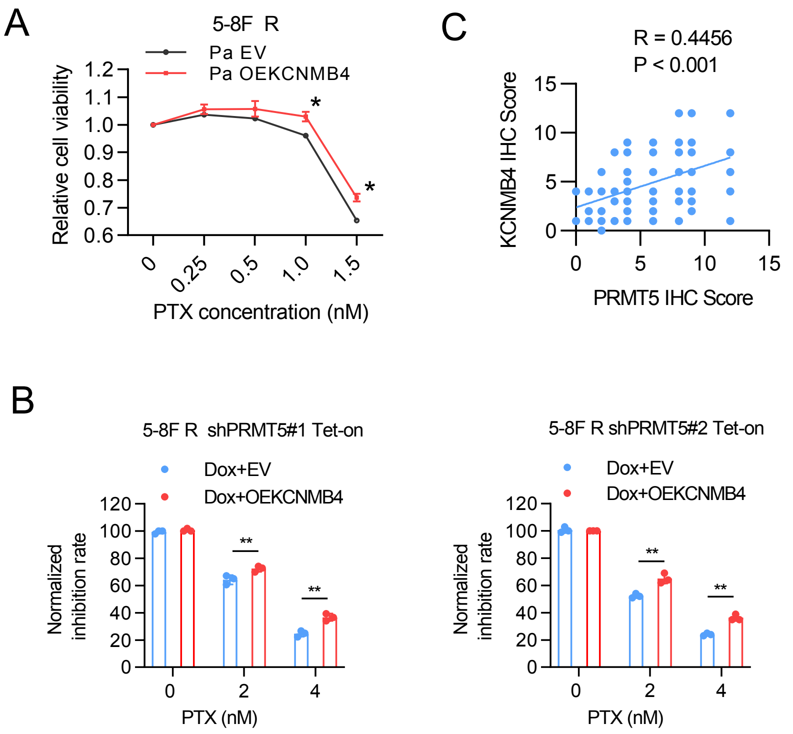

Supplementary Figure 4. Overexpression of KCNMB4 in 5-8F P cells and its expression profiles in publicly available data resources. A, Paclitaxel cytotoxicity assay of 5-8F R cells and 5-8F R cells with KCNMB4 overexpression. Cells were treated with different concentration of PTX for 96 h. Data are shown as means ± SD (n = 3). *P < 0.05. **B,** Rescue experiment of KCNMB4 in 5-8F R shPRMT5 tet-on cell line. 5-8F R shPRMT5 tet-on cells were transfected with empty vector or OE KCNMB4 plasmid and treated with both paclitaxel and Dox for 96 hours. Cell viability was measured by CellTiter Glo reagent and the inhibition rate of paclitaxel to nasopharyngeal carcinoma cells was calculated. Data are shown as means ± SD (n = 3). *P < 0.05; **P < 0.01. **C,** Pearson correlation between IHC scores of PRMT5 and KCNMB4 in the 194 patient samples with chemotherapy.

Supplementary Figure 5

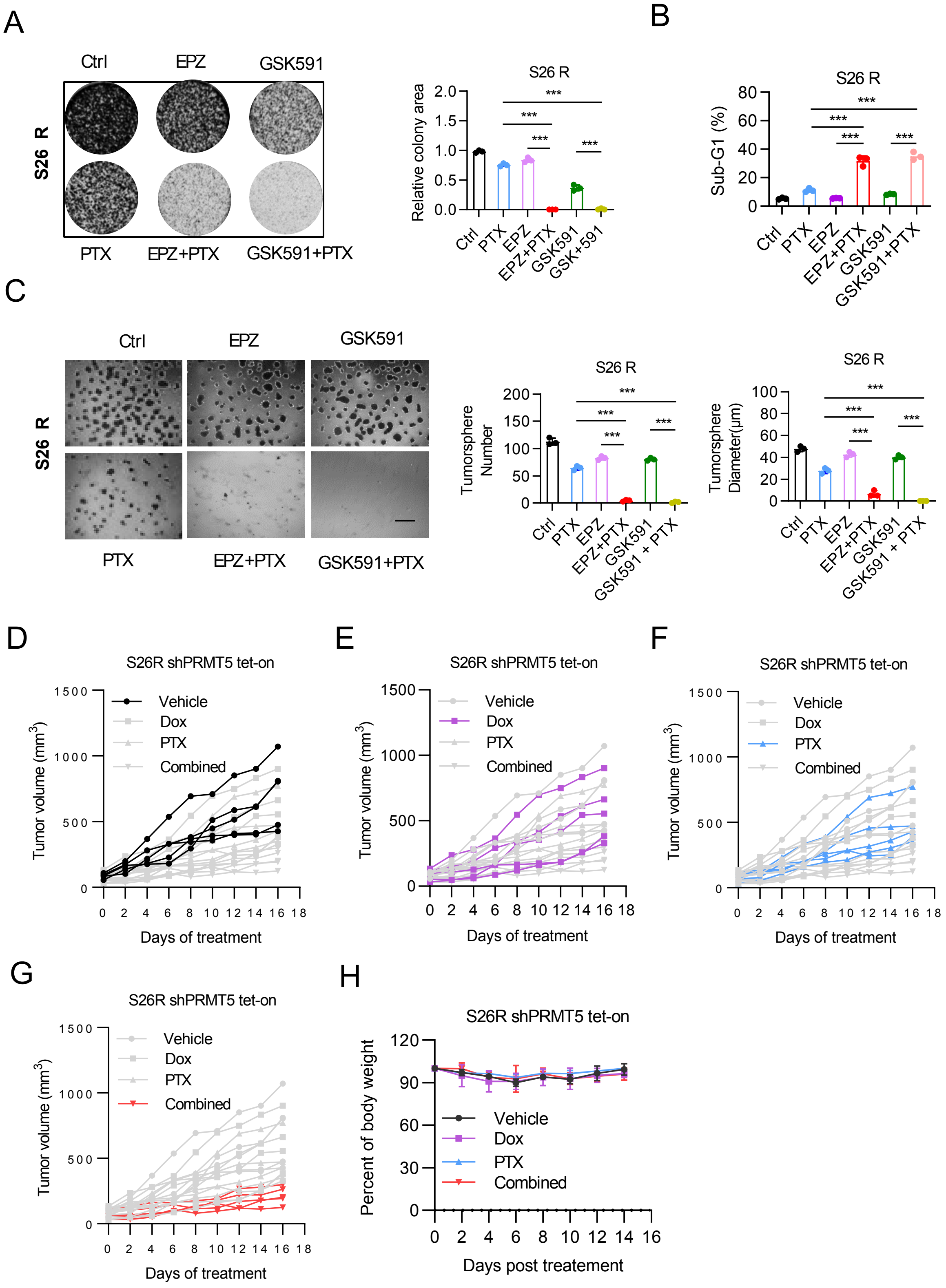

Supplementary Figure 5. PRMT5 downregulation restores the chemo-sensitivity of NPC. A, the effect of combined PRMT5 inhibitor and PTX treatment on S26R cell proliferation as shown by colony formation assay. EPZ015666 (EPZ) and GSK591 concentration used were 3 μmol/L. PTX concentration was 2nmol/mL. Left, representative images; Right, quantification. Data are shown as means ± SD (n = 3). ***P < 0.001. **B,** Sub-G1 population analysis in S26R cells treated with PRMT5 inhibitors, PTX or both for 96 h. Data are shown as means ± SD (n = 3). *P < 0.05; **P < 0.01; ***P < 0.001. **C,** Tumorsphere formation assay of S26R cells under PRMT5 inhibitor treatment with or without paclitaxel for 8 days. Representative images (left) and quantifications (right). Bars represent the means ± SD (n = 3). *, P < 0.05; **, P < 0.01; ***, P < 0.001. Scale bars, 200 μm. **D-G,** The growth curves of individual mice with different treatments. Error bars represent mean ± SEM (n = 5 per group). Doxycycline (Dox). **H,** Percentage Change in body weight of mice during the 16 days of treatment.
