# Supplementary material for: PRMT5 upregulates KCNMB4 expression via histone methylation to promote paclitaxel resistance in advanced nasopharyngeal carcinoma
Source: Cell Death Dis. 2026 Jan 9;17(1):19. doi: 10.1038/s41419-025-08190-y (PMC12789566; doi:10.1038/s41419-025-08190-y)
Supplement: Supplementary file 2 — Supplementary Tables S1-S7 [file 41419_2025_8190_MOESM2_ESM.pdf]

Supplementary Table 1. The sequence of siRNAs and shRNAs	
Gene name	Target sequence
siPRMT5#1	GGACCTGAGAGATGATATA
siPRMT5#2	CCAGAAGAGGAGAAGGATA
shKCNMB4#1	CCCAGCCATTTACTTGCTATT
shKCNMB4#2	GCTCGGCTTGTTTCTCATCAT
Tet-on shPRMT5#1	GGACCTGAGAGATGATATA
Tet-on shPRMT5#2	CCAGAAGAGGAGAAGGATA

Supplementary Table 2. The gene-specific primers used in qRT-PCR		
**Gene**	**Forward Primer 5'-3'**	**Reverse Primer 5'-3'**
18s	CGAACGTCTGCCCTATCAACTT	ACCCGTGGTCACCATGGTA
PRMT5	TGAAATTGGGGCTGACCTCC	CGGAAGATGAGCCTCTGGTG
KCNMB4	TGCTTCTGCATCGCACTCAT	GAACGCCCACCACAAATGTC
**Chip-qPCR primer**		
KCNMB4 P1 (-1310 ~ -1510)	ATGTGGAATATTGCGGGTCCA	ACTGTTCTCCAGACGACTCCT
KCNMB4 P2 (-530 ~ -410)	GTGTCGCTCTTCATCTTCGG	TCGAACACCTCGCCGATCT

Supplementary Table 3. Epigenetic drugs					
**Product name**	**M.W**	**Category**	**Drug target**	**Pathway**	**Index value**
Decitabine	228.21	DNA Methylt+D3:D52ransferase	DNA Methyltransferase inhibitor	Epigenetics	0.70
Azacitidine	244.2	DNA Methyltransferase	DNA Methyltransferase inhibitor	DNA Damage	0.68
Zebularine	228.2	DNA Methyltransferase	DNA Methyltransferase inhibitor	Epigenetics	0.74
Vorinostat (SAHA, MK0683)	264.3	Autophagy,HDAC	Autophagy,HDAC inhibitor	Endocrinology & Hormones	1.63
Panobinostat (LBH589)	349.43	HDAC	HDAC inhibitor	Epigenetics	1.07
Trichostatin A (TSA)	302.4	HDAC	HDAC inhibitor	Others	1.05
Entinostat (MS-275)	376.41	HDAC	HDAC (strongly inhibits HDAC1 and HDAC3)	Transmembrane Transporters	0.71
Belinostat (PXD101)	318.35	HDAC	HDAC inhibitor	Others	1.54
Pracinostat (SB939)	358.48	HDAC	Pan-HDAC inhibitor	Cytoskeletal Signaling	1.19
Pinometostat (EPZ5676)	562.71	Histone Methyltransferase	Protien methyltransferase DOT1L inhibitor	Epigenetics	0.81
GSK J4 HCl	453.96	Histone Demethylase	H3K27 histone demethylase JMJD3 and UTX inhibitor	Epigenetics	0.79
SGC 0946	618.57	Histone Methyltransferase	DOT1L methyltransferase inhibitor	Epigenetics	0.89
3-deazaneplanocin A (DZNeP) HCl	262.26	Histone Methyltransferase	S-adenosylhomocysteine hydrolase inhibitor	Epigenetics	0.76
C646	445.42	Histone Acetyltransferase	Histone acetyltransferase p300 inhibitor	Epigenetics	0.78
IOX1	189.17	Histone Demethylase	ALKBH5, 2OG oxygenases and JmjC demethylases inhibitor	Epigenetics	0.80
OG-L002	225.29	Histone Demethylase	LSD1 inhibitor	Epigenetics	0.84
MM-102	669.8	Histone Methyltransferase	WDR5/MLL1 protein-protein interaction inhibitor	Epigenetics	0.89
JIB-04	308.76	Histone Demethylase	Jumonji histone demethylase pan-selective inhibitor	Epigenetics	1.99
PFI-2 HCl	499.52	Histone Methyltransferase	Lysine methyltransferase SETD7 inhibitor	Epigenetics	0.62
ML324	349.43	Histone Demethylase	Jumonji histone demethylase (JMJD2) inhibitor	Epigenetics	0.65
EPZ004777	539.67	Histone Methyltransferase	DOT1L inhibitor	Epigenetics	0.80
MG149	340.46	Histone Acetyltransferase	Histone acetyltransferase inhibitor	Epigenetics	0.85
UNC0379	413.56	Histone Methyltransferase	N-lysine methyltransferase SETD8 inhibitor	Epigenetics	0.62
A-366	329.44	Histone Methyltransferase	G9a/GLP histone lysine methyltransferase inhibitor	Epigenetics	0.75
GSK-LSD1 2HCl	289.24	Histone Demethylase	LSD1 inhibitor	Epigenetics	0.73
GSK J1	389.45	Histone Demethylase	H3K27 histone demethylase inhibitor	Epigenetics	0.74
Anacardic Acid	348.52	Histone Acetyltransferase	p300 and p300/CBP-associated factor histone acetyltranferases inhibitor	Epigenetics	0.74
BRD4770	413.47	Histone Methyltransferase	Histone methyltransferase G9a inhibitor,	Epigenetics	0.81
UNC0631	635.93	Histone Methyltransferase	Histone methyltransferase G9a inhibitor	Epigenetics	0.70
EI1	390.48	Histone Methyltransferase	EZH2 inhibitor	Epigenetics	0.83
CPI-169	528.66	Histone Methyltransferase	EZH2 inhibitor	Epigenetics	0.86
MI-2 (Menin-MLL Inhibitor)	375.55	Histone Methyltransferase	Menin-MLL interaction inhibitor	Epigenetics	0.80
CPI-360	437.53	Histone Methyltransferase	EZH2 inhibitor	Epigenetics	0.81
SP2509	437.90	Histone Demethylase	LSD1 inhibitor	Epigenetics	0.75
EPZ015666(GSK3235025)	383.44	Histone Methyltransferase	PRMT5 inhibitor	Epigenetics	0.54
ORY-1001 (RG-6016) 2HCl	303.27	Histone Demethylase	Lysine-specific demethylase LSD1/KDM1A inhibitor	Epigenetics	0.81
GSK2879552 2HCl	437.4	Histone Demethylase	LSD1 inhibitor	Epigenetics	0.77
GSK503	526.67	Histone Methyltransferase	EZH2 methyltransferase inhibitor	Epigenetics	0.68
EPZ011989	642.27	Histone Methyltransferase	EZH2 inhibitor	Epigenetics	0.77
SGC707	298.34	Histone Methyltransferase	PRMT3 inhibitor	Epigenetics	0.79
BIX 01294	600.02	Histone Methyltransferase	G9a inhibitor	Epigenetics	0.66
GSK591	380.48	Histone Methyltransferase	PRMT5 inhibitor	Epigenetics	0.57
MS023	287.40	Histone Methyltransferase	Type I PRMT inhibitor (PRMT1, PRMT3, PRMT4,PRMT6, PRMT8)	Epigenetics	0.59
HLCL-61 HCL	380.91	Histone Methyltransferase	Type I PRMT inhibitor (PRMT1, PRMT3, PRMT4,PRMT6, PRMT8)	Epigenetics	0.79
SRT1720 HCl	506.02	Sirtuin	SIRT1 activator	Others	0.82
PFI-1 (PF-6405761)	347.39	Epigenetic Reader Domain	BET (bromodomain-containing protein) inhibitor	Epigenetics	0.77
Selisistat (EX 527)	248.71	Sirtuin	SIRT1 inhibitor	Epigenetics	0.84
Sirtinol	394.47	Sirtuin	SIRT1 inhibitor	Epigenetics	0.83
(+)-JQ1	456.99	Epigenetic Reader Domain	BET bromodomain inhibitor	Epigenetics	0.64
I-BET-762	423.9	Epigenetic Reader Domain	BET protein inhibitor	Epigenetics	0.73
SGC-CBP30	509.04	Epigenetic Reader Domain	CREBBP/EP300 inhibitor	Epigenetics	0.93
RVX-208	370.4	Epigenetic Reader Domain	BET bromodomain inhibitor	Epigenetics	0.82
CPI-203	399.9	Epigenetic Reader Domain	BET bromodomain inhibitor	Epigenetics	0.64
MS436	383.42	Epigenetic Reader Domain	BET bromodomain inhibitor	Epigenetics	0.81
PFI-3	321.37	Epigenetic Reader Domain	SMARCAand PBI bromodomain inhibitor	Epigenetics	0.75
OTX015	491.99	Epigenetic Reader Domain	BET bromodomain inhibitor	Epigenetics	0.68
GSK1324726A (I-BET726)	434.91	Epigenetic Reader Domain	BET family protein inhibitor	Epigenetics	0.63
OF-1	440.31	Epigenetic Reader Domain	BRPF1B and BRPF2 bromodomain inhibitor	Epigenetics	0.74
I-BRD9	497.55	Epigenetic Reader Domain	BRD9 inhibitor	Epigenetics	0.79
PF-CBP1 HCl	525.08	Epigenetic Reader Domain	CREB-binding protein(CREBBP) bromodomain inhibitor	Epigenetics	0.80
CUDC-101	434.49	EGFR,HDAC,HER2	EGFR,HDAC,HER2 inhibitor	Epigenetics	0.80
Resminostat	349.4	HDAC	HDAC inhibitor	Epigenetics	0.60
Ricolinostat (ACY-1215)	433.5	HDAC	HDAC6 inhibitor	Epigenetics	0.63
Resveratrol	228.24	Autophagy,Sirtuin	Autophagy,Sirtuin inhibitor	Epigenetics	0.78
Entacapone	305.29	Histone Methyltransferase	Catechol-O-methyltransferase(COMT) inhibitor	Epigenetics	0.76
Tranylcypromine (2-PCPA) HCl	169.65	MAO	Monoamine oxidase inhibitor	Epigenetics	0.81
Tazemetostat (EPZ-6438)	572.74	Histone Methyltransferase	EZH2 inhibitor	Epigenetics	0.82

Supplementary Table 4 Patient demographics and clinical characteristics (PRMT5)

Characteristic	PRMT5	
	**Low**	**High**
Total	119(61.4)	75(38.6)
**Age, years**		
Median (range)	47(21–67)	48(21–77)
＞45	54(45.3）	36(48.0)
≤45	65(54.7)	39(52.0)
**Sex**		
Female	79(66.4)	54(72.0)
Male	40(33.6)	21(28.0)
**WHO Pathologic type**		
I	0(0.0)	0(0.0)
II	1(0.8)	0(0.0)
III	118(100)	75(100)
**T stage***		
T1	7(5.9)	2(2.7)
T2	22(18.5)	13(17.3)
T3	67(56.3)	41(54.7)
T4	23(19.3)	19(25.3)
**N stage***		
N0	5(4.2)	4(5.3)
N1	55(46.2)	26(34.7)
N2	39(32.8)	36(48.0)
N3	20(16.8)	9(12.0)
**AJCC stage***		
I	0(0.0)	0(0.0)
II	13(11.0)	8(10.6)
III	65(54.6)	41(54.7)
IV	21(17.6)	17(22.7)
V	20(16.8)	9(12.0)
**EBV DNA(copies/mL)**		
<1500	66(55.5)	26(34.7)
≥1500	53(44.5)	49(65.3)
**Chemo-radiotherapy#**		
Yes	117(98.3)	72(96.0)
No	2(1.7)	3(4.0)

WHO, World Health Organization; TNM, tumor－node－metastases; EBV, Epstein－Barr virus; LDH, serum lactate dehydrogenase;

*According to the 8th edition of UICC/AJCC staging system; #Chemodrugs used were:carboplatin, cisplatin, paclitaxel, 5-FU, gemcitabine and so on. These agents were administered in combination, typically using two or three drugs concurrently.

Supplementary Table 5 Multivariable analysis of prognostic factors of the NPC patients (PRMT5)

Characteristics	HR[95%CI]	P value
**PRMT5**	3.26 [1.68, 6.33]	<0.001
<8		
≥8		
**Age**	1.22 [0.64, 2.34]	0.547
<45		
≥45		
**T**	0.81 [0.38, 1.74]	0.595
T1-T2		
T3-T4		
**N**	1.74 [0.86, 3.55]	0.125
N0-N1		
N2-N3		
**Stage**	1.22 [0.63, 2.35]	0.559
I-II		
III-IV		
**EBV DNA**	2.15 [1.03, 4.49]	0.041
<1500		
≥1500		
**Regimen**	1.15 [0.70, 1.88]	0.59
0		
1		
2		

EBV, Epstein－Barr virus; Hazard ratios (HR) and p-values were calculated using an adjusted multivariate Cox proportional hazards regression model.

Supplementary Table 6 Patient demographics and clinical characteristics (KCNMB4)

**Characteristic**	**KCNMB4**	
	**Low**	**High**
Total	112(57.8)	82(42.2)
**Age, years**		
Median (range)	47(21–67)	48(21–77)
＞45	60(53.6)	44(53.7)
⩽45	52(46.4）	38(43.6)
**Sex**		
Female	74(60.1)	59(71.9)
Male	38(33.9)	23(28.0)
**WHO Pathologic type**		
I	0(0.0)	0(0.0)
II	1(0.9)	0(0.0)
III	111(99.1)	82(100)
**T stage***		
T1	7(6.3)	2(2.4)
T2	23(20.5)	12(14.6)
T3	58(51.8)	50(61.0)
T4	24(21.4)	18(22.0)
N stage*		
N0	6(5.3)	3(3.7)
N1	49(43.8)	32(39.0)
N2	40(35.7)	35(42.7)
N3	17(15.2)	12(14.6)
**AJCC stage***		
I	0(0.0)	0(0.0)
II	14(12.5)	7(8.5)
III	58(51.8)	48(58.5)
IV	23(20.5)	15(18.4)
V	17(15.2)	12(14.6)
**EBV DNA(copies/mL)**		
<1500	57(50.9)	35(42.7)
⩾1500	55(49.1)	47(57.3)
**Chemo-radiotherapy**[#]		
Yes	108(96.4)	81(98.7)
No	4(3.6)	1(1.3)

WHO, World Health Organization; TNM, tumor–node–metastases; EBV, Epstein–Barr virus; LDH, serum lactate dehydrogenase;

*According to the 8th edition of UICC/AJCC staging system; [#]Chemodrugs used were:carboplatin, cisplatin, paclitaxel, 5-FU, gemcitabine and so on. These agents were administered in combination, typically using two or three drugs concurrently.

Supplementary Table 7 Multivariable analysis of prognostic factors of the NPC patients (KCNMB4)

Characteristics	HR[95%CI]	P value
**KCNMB4**	2.58 [1.35, 4.90]	0.004
<6		
≥6		
**Age**	1.15 [0.61, 2.15]	0.672
<45		
≥45		
**T**	0.92 [0.44, 1.95]	0.83
T1-T2		
T3-T4		
**N**	1.62 [0.79, 3.31]	0.188
N0-N1		
N2-N3		
**Stage**	1.36 [0.70, 2.64]	0.368
I-II		
III-IV		
**EBV DNA**	2.49 [1.19, 5.21]	0.016
<1500		
≥1500		
**Regimen**	0.93 [0.57, 1.51]	0.766
0		
1		
2		

EBV, Epstein－Barr virus; Hazard ratios (HR) and p-values were calculated using an adjusted multivariate Cox proportional hazards regression model.
