# Supplementary material for: PRMT5 upregulates KCNMB4 expression via histone methylation to promote paclitaxel resistance in advanced nasopharyngeal carcinoma
Source: Cell Death Dis. 2026 Jan 9;17(1):19. doi: 10.1038/s41419-025-08190-y (PMC12789566; doi:10.1038/s41419-025-08190-y)

Figure 2 uncut Western gel

F

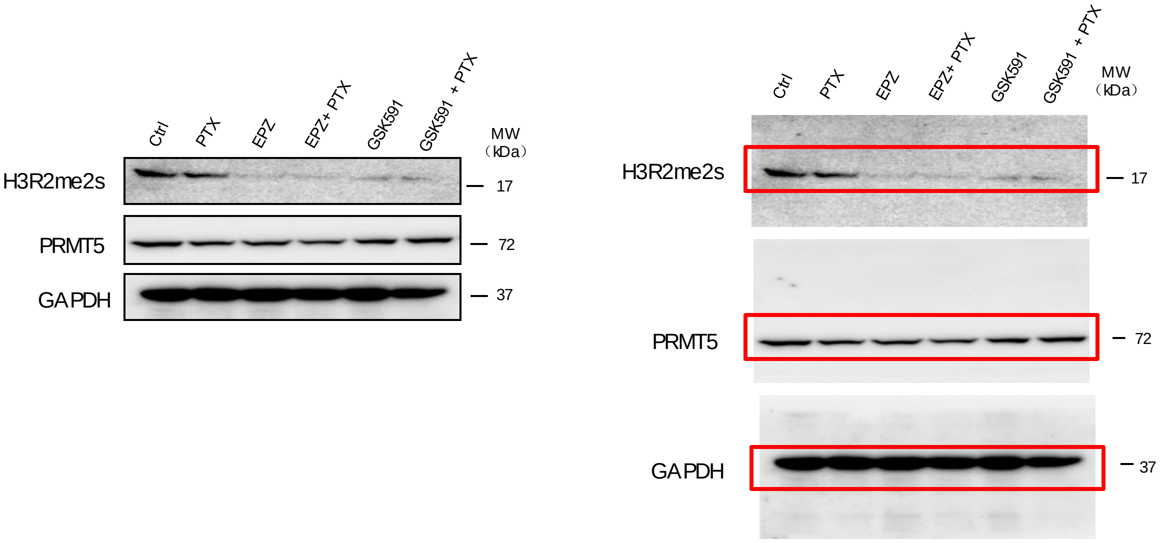

Figure 3 uncut Western gel

A

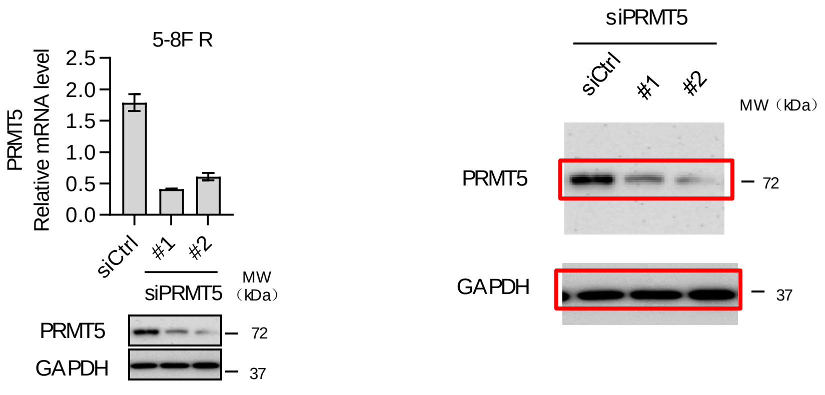

D

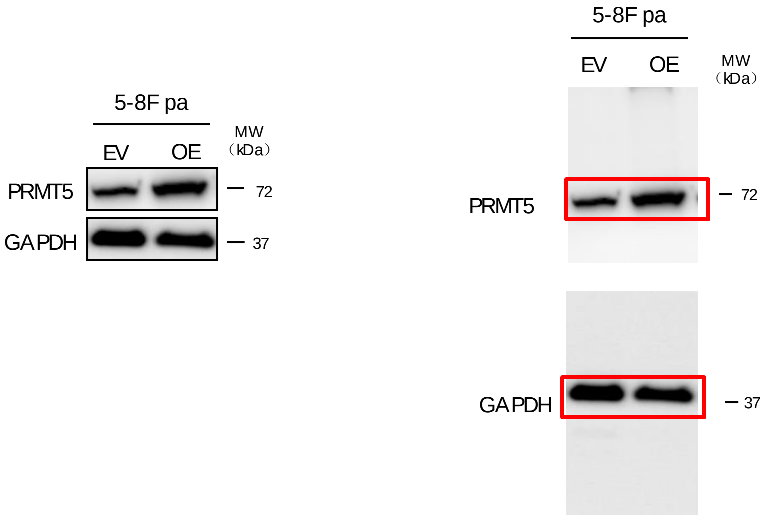

Figure 4 uncut Western gel

C

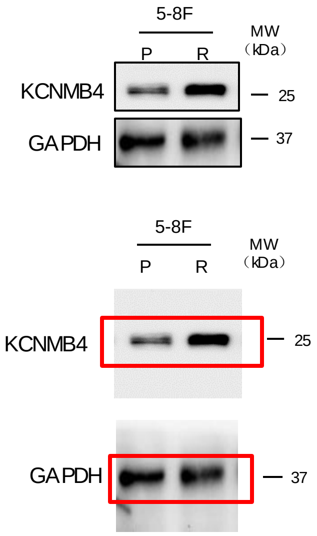

D

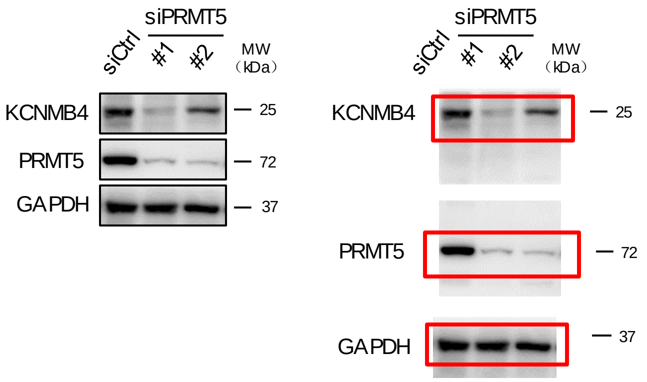

E

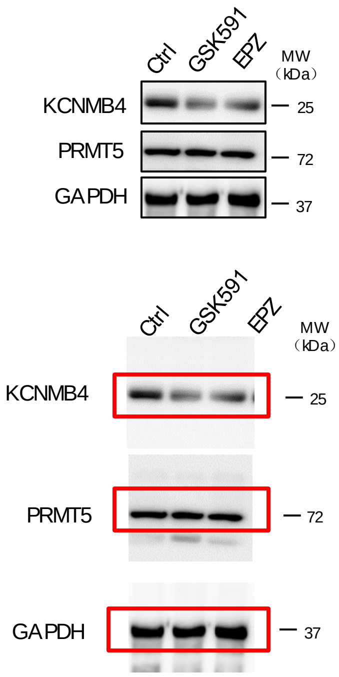

H

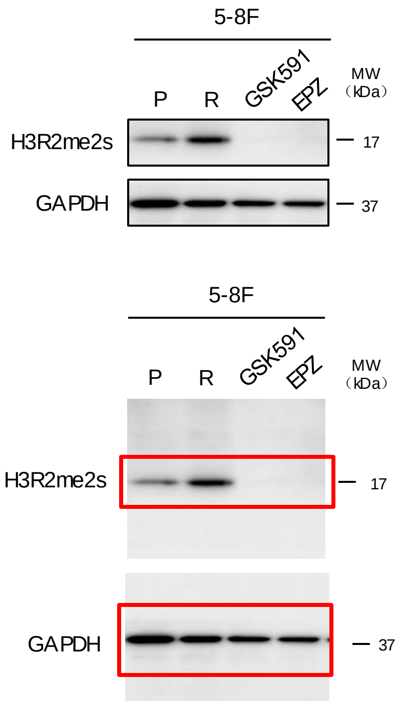

Figure 5 uncut Western gel

A

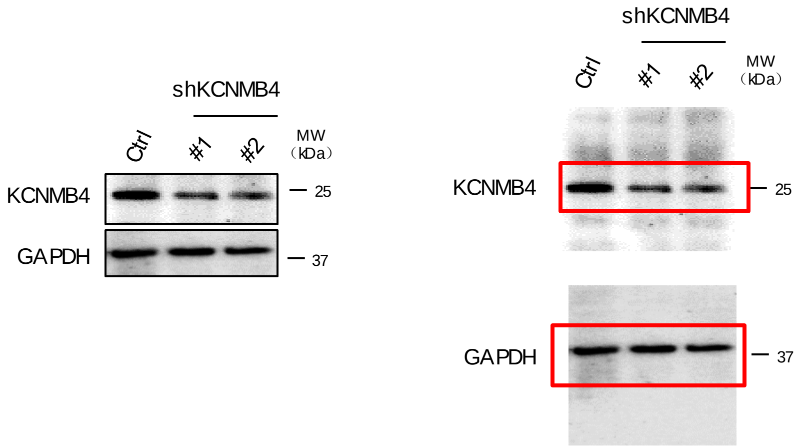

C

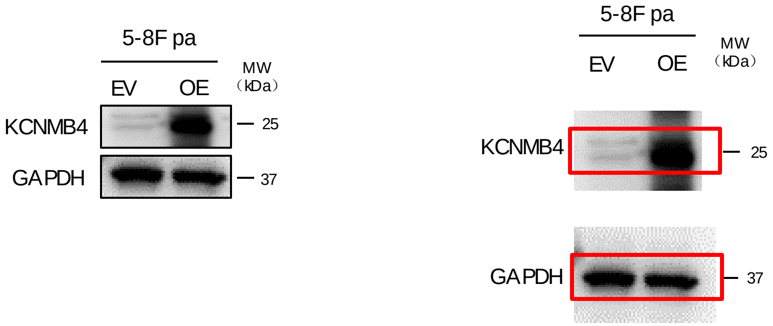

Supplementary Figure 2 uncut Western gel

A

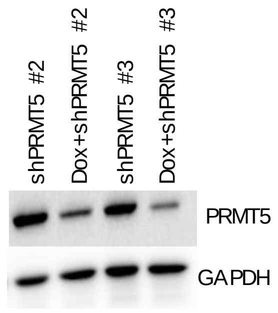

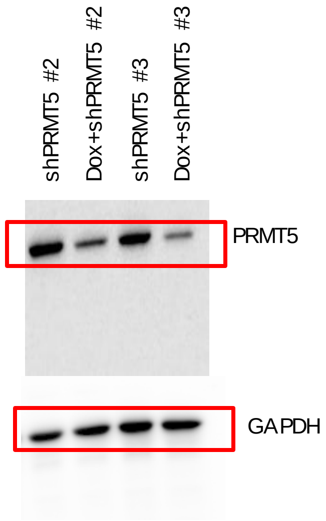

B

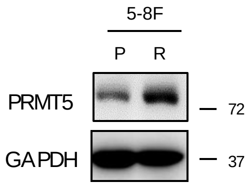

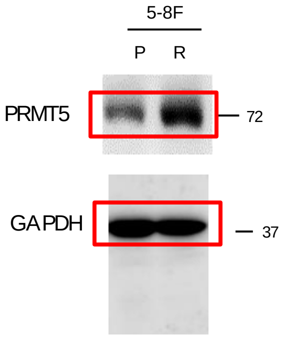

Supplement: Supplementary file 3 — Supplementary uncut Western blot [file 41419_2025_8190_MOESM3_ESM.pdf]
