# Supplementary material for: PRMT5 upregulates KCNMB4 expression via histone methylation to promote paclitaxel resistance in advanced nasopharyngeal carcinoma
Source: Cell Death Dis. 2026 Jan 9;17(1):19. doi: 10.1038/s41419-025-08190-y (PMC12789566; doi:10.1038/s41419-025-08190-y)

Original stained images and colony assay plates

Figure 2

A

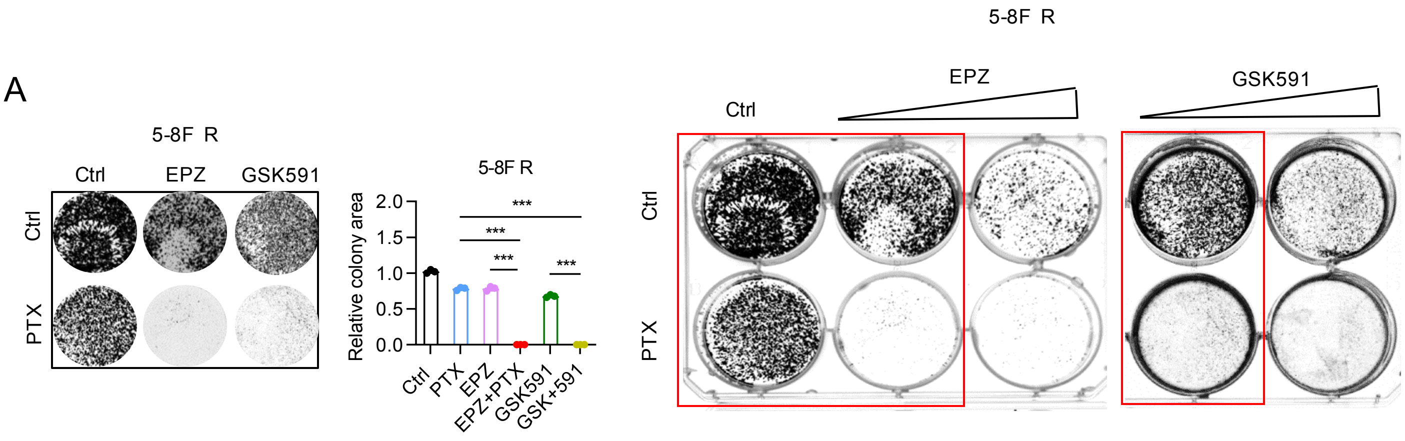

Figure 3

A

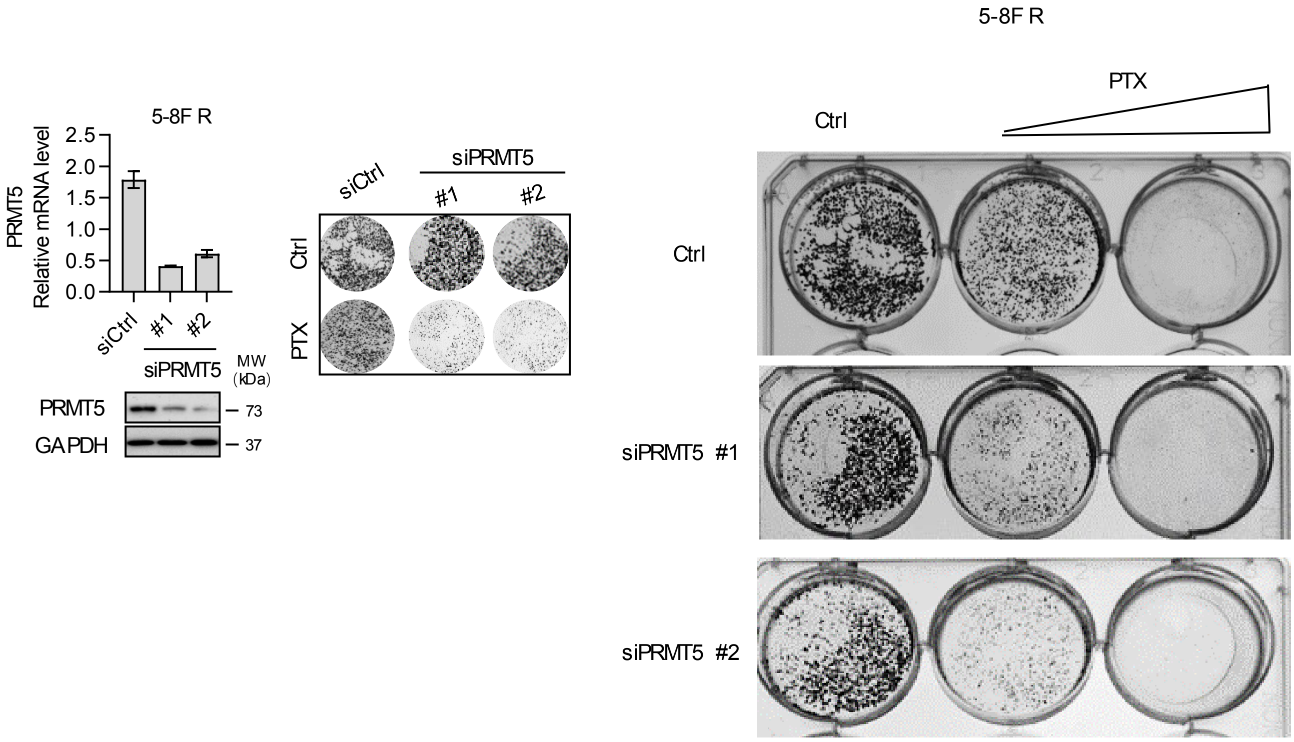

Figure 3

E

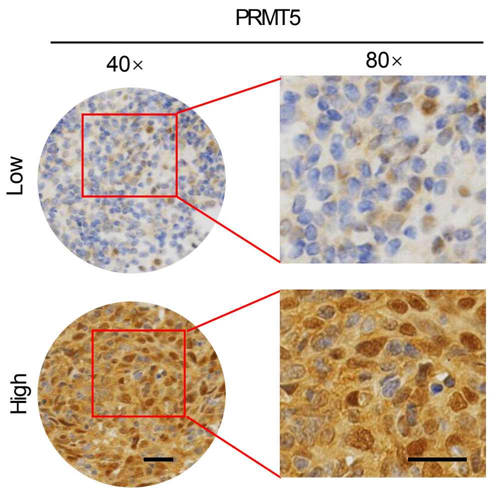

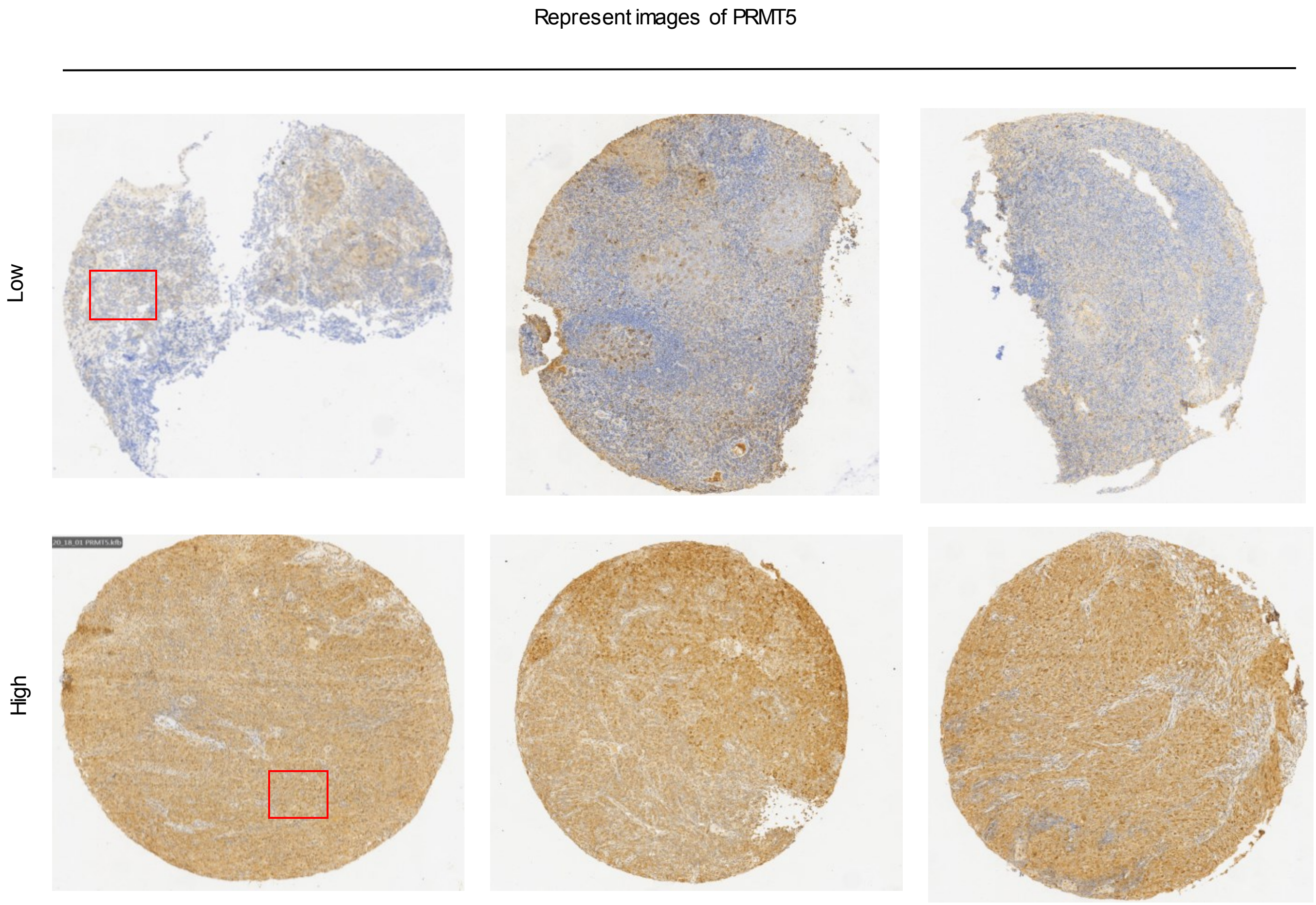

Figure 5

D

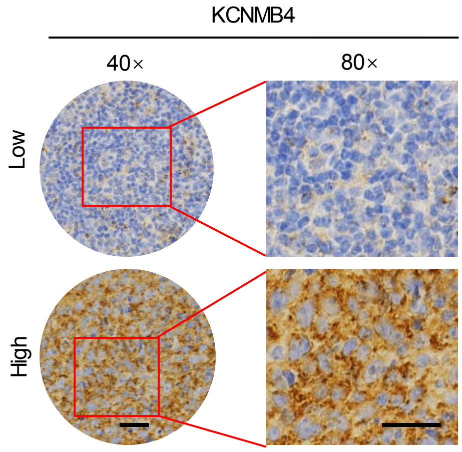

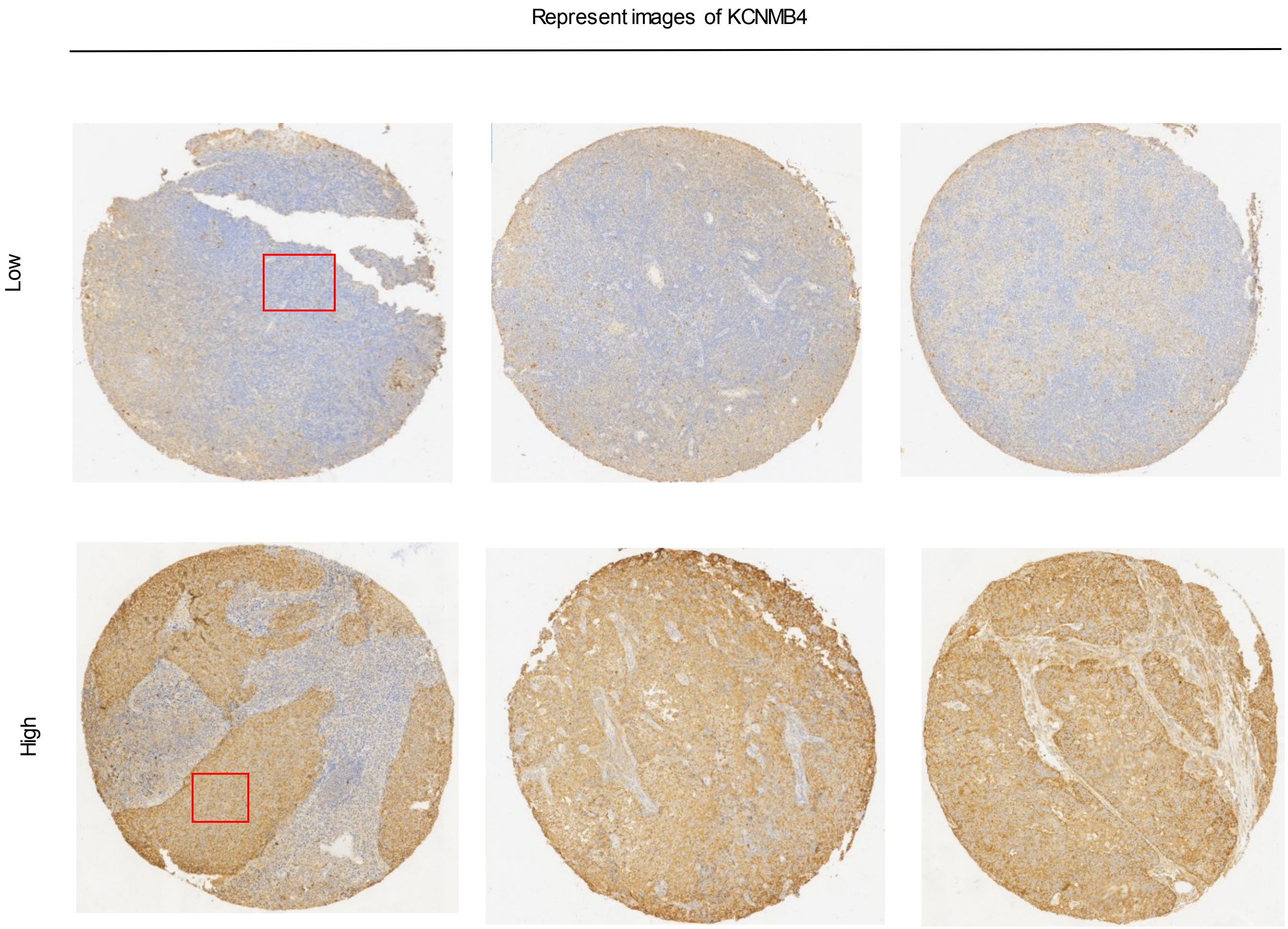

Supplementary Figure 1

B

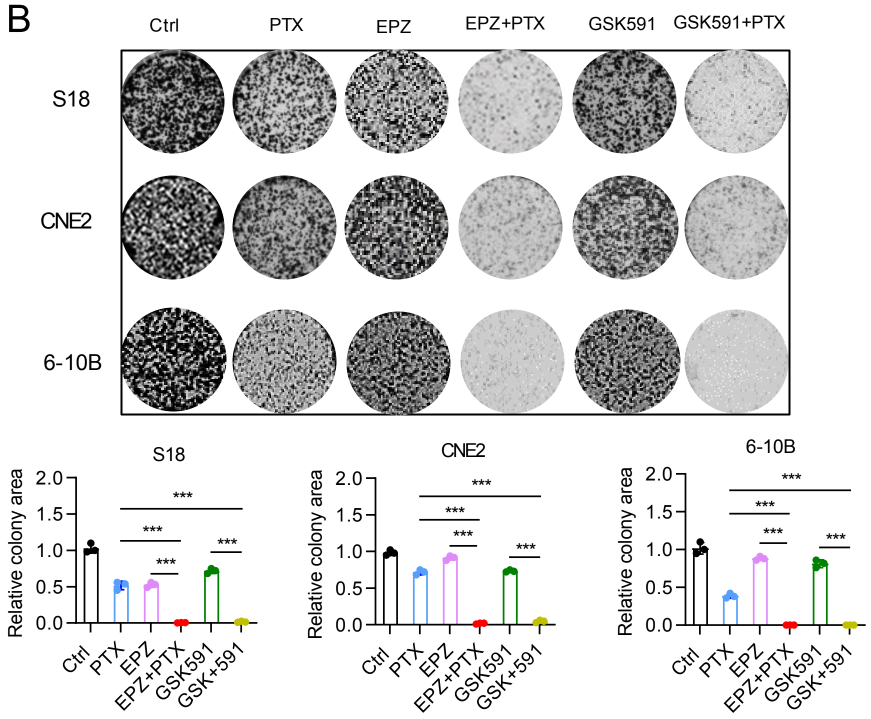

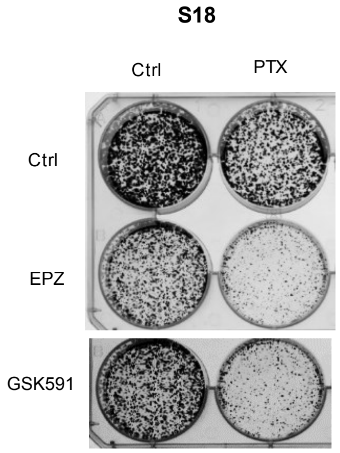

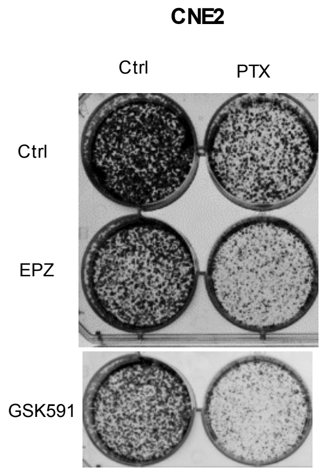

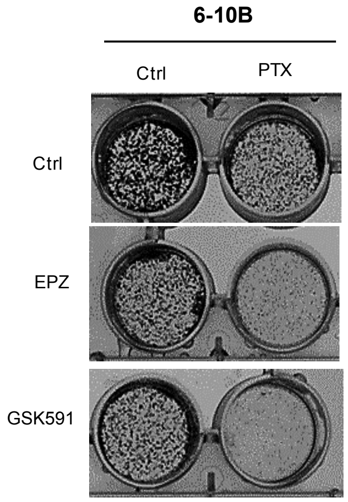

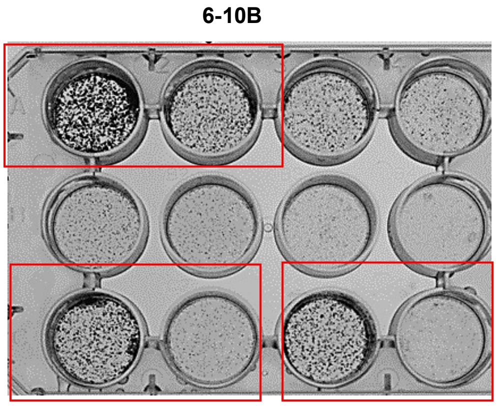

Supplementary Figure 1

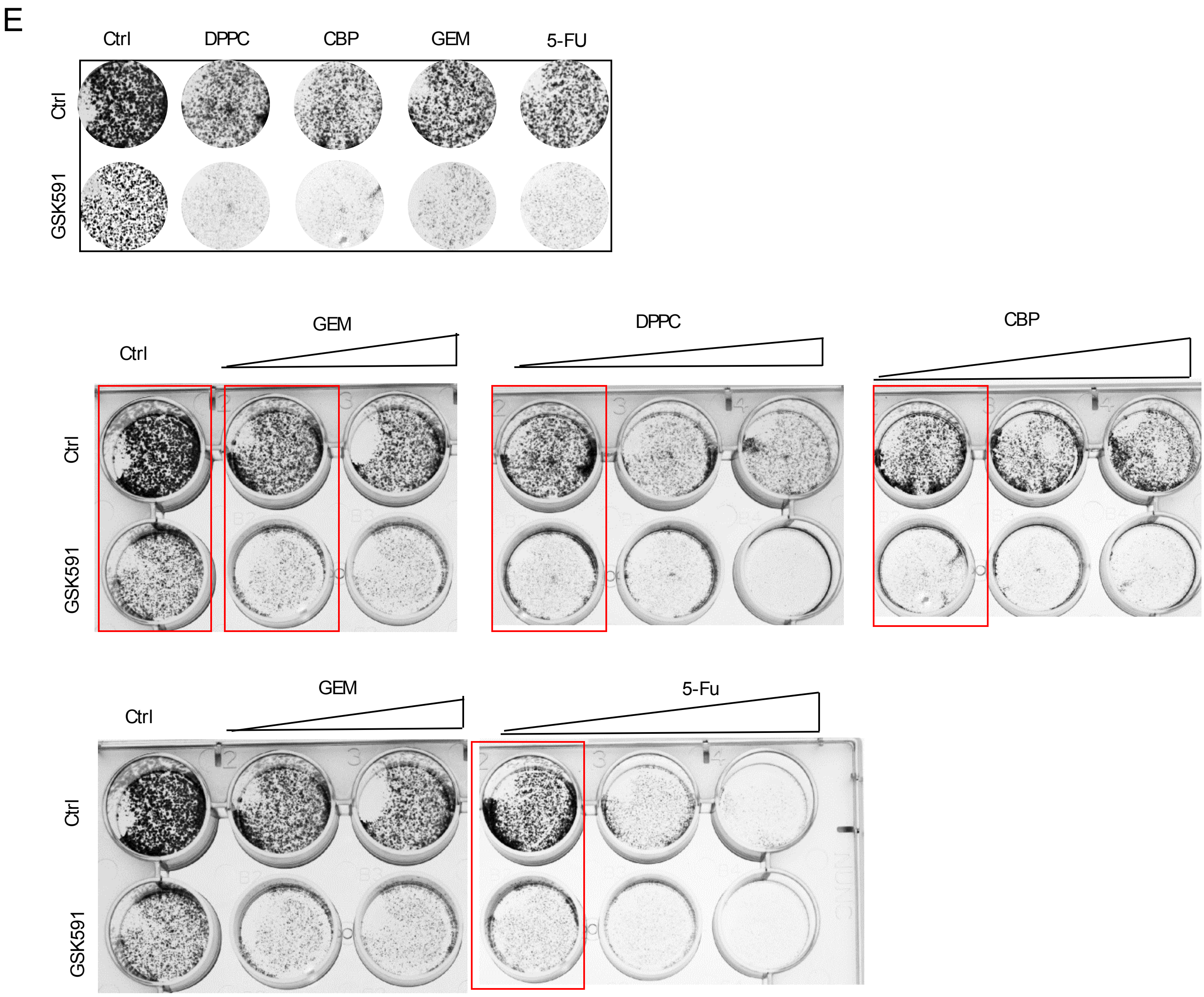

Supplementary Figure 5

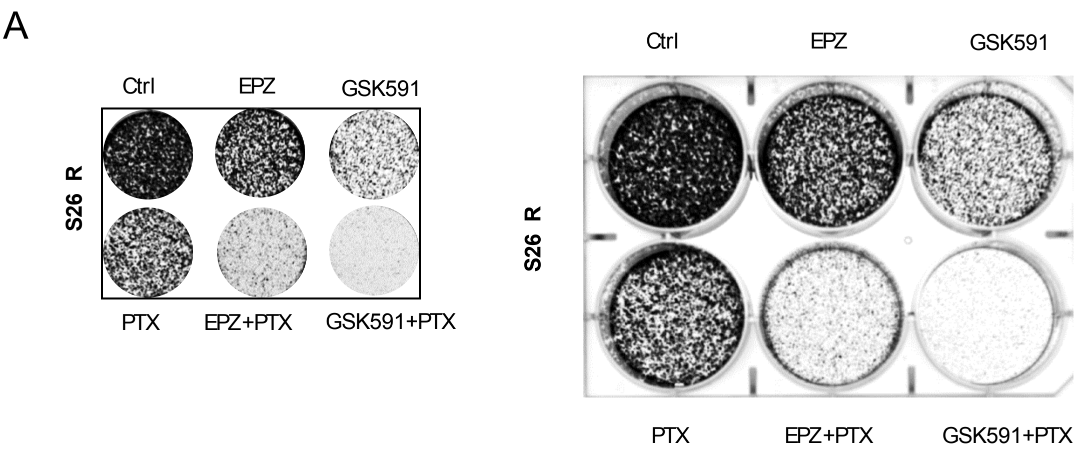

Supplement: Supplementary file 4 — Supplementary raw data [file 41419_2025_8190_MOESM4_ESM.pdf]
